# Precision Metrics: A Narrative Review on Unlocking the Power of KPIs in Radiology for Enhanced Precision Medicine

**DOI:** 10.3390/jpm14090963

**Published:** 2024-09-10

**Authors:** Andrea Lastrucci, Yannick Wandael, Angelo Barra, Vittorio Miele, Renzo Ricci, Lorenzo Livi, Graziano Lepri, Rosario Alfio Gulino, Giovanni Maccioni, Daniele Giansanti

**Affiliations:** 1 Department of Allied Health Professions, Azienda Ospedaliero-Universitaria Careggi, 50134 Florence, Italy; andrea.lastrucci@unifi.it (A.L.); wandaely@aou-careggi.toscana.it (Y.W.); barraa@aou-careggi.toscana.it (A.B.); riccire@aou-careggi.toscana.it (R.R.); 2 Department of Emergency Radiology, Careggi University Hospital, Largo Brambilla 3, 50134 Florence, Italy; vmiele@sirm.org; 3 Department of Experimental and Clinical Biomedical Sciences “M. Serio”, University of Florence, 50134 Florence, Italy; lorenzo.livi@unifi.it; 4 Azienda Unità Sanitaria Locale Umbria 1, Via Guerriero Guerra 21, 06127 Perugia, Italy; graziano.lepri@uslumbria1.it; 5 Facoltà di Ingegneria, Università di Tor Vergata, Via del Politecnico, 1, 00133 Rome, Italy; gulino@disp.uniroma2.it; 6 Centro Nazionale TISP, Istituto Superiore di Sanità, Viale Regina Elena 299, 00161 Rome, Italy; giovanni.maccioni@iss.it

**Keywords:** radiology, KPI, key performance indicators, personalized medicine, precision medicine, metrics, artificial intelligence

## Abstract

(*Background*) Over the years, there has been increasing interest in adopting a quality approach in radiology, leading to the strategic pursuit of specific and key performance indicators (KPIs). These indicators in radiology can have significant impacts ranging from radiation protection to integration into digital healthcare. (*Purpose*) This study aimed to conduct a narrative review on the integration of key performance indicators (KPIs) in radiology with specific key questions. (*Methods*) This review utilized a standardized checklist for narrative reviews, including the ANDJ Narrative Checklist, to ensure thoroughness and consistency. Searches were performed on PubMed, Scopus, and Google Scholar using a combination of keywords related to radiology and KPIs, with Boolean logic to refine results. From an initial yield of 211 studies, 127 were excluded due to a lack of focus on KPIs. The remaining 84 studies were assessed for clarity, design, and methodology, with 26 ultimately selected for detailed review. The evaluation process involved multiple assessors to minimize bias and ensure a rigorous analysis. (*Results and Discussion*) This overview highlights the following: KPIs are crucial for advancing radiology by supporting the evolution of imaging technologies (e.g., CT, MRI) and integrating emerging technologies like AI and AR/VR. They ensure high standards in diagnostic accuracy, image quality, and operational efficiency, enhancing diagnostic capabilities and streamlining workflows. KPIs are vital for radiological safety, measuring adherence to protocols that minimize radiation exposure and protect patients. The effective integration of KPIs into healthcare systems requires systematic development, validation, and standardization, supported by national and international initiatives. Addressing challenges like CAD-CAM technology and home-based radiology is essential. Developing specialized KPIs for new technologies will be key to continuous improvement in patient care and radiological practices. (*Conclusions*) In conclusion, KPIs are essential for advancing radiology, while future research should focus on improving data access and developing specialized KPIs to address emerging challenges. Future research should focus on expanding documentation sources, improving web search methods, and establishing direct connections with scientific associations.

## 1. Introduction

### 1.1. Diagnostic and Interventional Radiology: An Overview

Diagnostic and interventional radiology is a sophisticated branch of medicine that merges diagnostic imaging and therapeutic procedures guided by radiological techniques [1,2,3,4]. Modern diagnostic imaging includes methods such as X-rays, ultrasound, magnetic resonance imaging (MRI), and Positron Emission Tomography (PET) [1,2,3]. These technologies provide detailed internal images, enhancing the accuracy of diagnoses and the effectiveness of treatments.

*Impact on Personalized and Precision Medicine*

In *personalized medicine*, diagnostic radiology allows for tailored healthcare by offering detailed, patient-specific imaging that enables precise diagnoses and customized treatment plans. For instance, MRI and PET scans can uncover specific characteristics of a patient’s condition, facilitating targeted therapies that reduce side effects and enhance treatment efficacy [4,5].

*Precision medicine* benefits from radiology’s ability to identify and monitor disease markers at a molecular level. Techniques such as MRI and PET enable the early and precise detection of diseases, allowing for treatments that are adjusted based on real-time imaging data. This precision optimizes patient outcomes by adapting therapies to the disease’s response [6,7].

*Team Collaboration and Procedures*

The field relies on a multidisciplinary team, including radiologists, interventional radiologists, radiologic technologists, specialized nurses, physiatrists, physical therapists, oncologists, and vascular surgeons. This collaboration ensures comprehensive patient care through less invasive procedures like angiography, ultrasound, MRI, CT scans, and PET scans. These techniques reduce the need for general anesthesia, shorten recovery times, and minimize intervention risks [8,9,10].

Overall, diagnostic and interventional radiology significantly enhances both personalized and precision medicine by providing detailed imaging and enabling tailored, effective treatments. The integration of these technologies into healthcare practices improves clinical outcomes and patient quality of life through less invasive, targeted interventions [1,2,3,4,5,6,7,8,9,10].

### 1.2. Advancing Healthcare: Integrating Radiology with Cutting-Edge Technologies

Emerging technologies such as artificial intelligence (AI), augmented and virtual reality (AR/VR), advanced functional imaging, 3D printing, photon-counting CT scanners, liquid biopsy, radiomics, molecular imaging, nanotechnologies, portable devices, and digital twins are transforming radiology [11,12]. These innovations enhance diagnostic accuracy and speed, facilitating early intervention and allowing for treatments tailored to each patient’s unique profile.

*Impact on Personalized Medicine:* By enabling detailed, patient-specific imaging, these technologies support the customization of medical treatments. AI and advanced imaging techniques provide precise information about individual disease characteristics, leading to interventions that are specifically designed to meet each patient’s needs, thereby improving treatment outcomes and reducing side effects.

*Impact on Precision Medicine:* Technologies like 3D printing and digital twins contribute to precision medicine by creating highly accurate models for diagnosis and treatment planning. These tools ensure that therapies are targeted with exceptional precision, enhancing their effectiveness and minimizing potential risks.

Overall, these advancements not only accelerate the diagnostic process and improve imaging quality but also enhance the capabilities of healthcare professionals. They integrate patient-specific data into clinical decision-making, support safer and more effective interventions, and improve educational tools for medical professionals through immersive visualization [13].

### 1.3. Elevating Radiology Quality: Integrating Key Performance Indicators for Precision Medicine

#### 1.3.1. Advancements in Radiology and Their Impact on Quality

The rapid evolution of radiology, particularly its growing role in precision and personalized medicine, is fundamentally reshaping healthcare quality. This transformation emphasizes the need for a comprehensive approach to quality, encompassing all aspects of radiological practice—procedures, workflow organization, and patient care [14]. By integrating advanced technologies and establishing robust quality measures, radiology is positioned to significantly enhance patient outcomes, safety, and overall healthcare efficiency [15]. 

*Advancements in Radiology and Their Impact on Quality:* The integration of cutting-edge technologies such as artificial intelligence (AI), molecular imaging, and advanced medical devices has revolutionized the field [16,17]. These innovations not only improve diagnostic accuracy and early intervention but also enable the development of individualized treatment plans. This shift towards precision medicine facilitates the delivery of more effective and less invasive treatments, ultimately enhancing patient care quality [17]. A holistic quality approach in radiology also focuses on optimizing operational efficiencies, reducing patient wait times, and improving the overall patient experience. These efforts ensure timely diagnoses and treatments, adherence to safety protocols, and a continuous commitment to excellence in patient care. 

*The Growing Focus on Quality in Radiology Research:* Over the years, there has been a significant increase in research dedicated to quality in radiology, reflecting the field’s evolution. A PubMed search using the key phrase in Box S1 reveals 69,056 studies published since 1909, highlighting the extensive scholarly interest in radiology. Specifically, 6732 studies focusing on quality in radiology have been published since 1958 (this period marks a significant milestone, occurring decades after the establishment of independent nonprofit organizations that began offering hospital accreditation starting from the 1950s [18]), comprising approximately 9.74% of all radiological research (Figure S1). 

A noteworthy observation is the accelerated interest in both general radiology and quality-focused radiology over the past five years, a period notably shaped by the global pandemic. This period has seen a marked increase in research output (Figures S2 and S3):

- In the broader field of radiology, studies conducted in the past five years account for 32.3% of the total volume (Figure S2).
- Concurrently, within the subset of radiology studies with a specific emphasis on quality, there has been a proportional rise, with these studies comprising 35.2% of the total research output (Figure S3).

Trends in the past five years show similar patterns, albeit with a slight proportional increase of 2.9% in the interest in studies focusing on quality in radiology. These trends underscore a growing recognition of the importance of quality standards in radiology.

This growing focus, particularly over the past five years, underscores the recognition of quality standards as a critical component of radiological practice, driven by technological advancements and the evolving healthcare landscape. 

#### 1.3.2. The Role of Key Performance Indicators (KPIs) in Ensuring Radiology Quality

Key Performance Indicators (KPIs): KPIs are measurable values that demonstrate how effectively an organization is achieving its key business objectives (KPI definition) [19]. In the context of radiology, KPIs are used to assess various aspects of performance including diagnostic accuracy, operational efficiency, patient satisfaction, and adherence to safety protocols. They provide actionable insights that help in improving the quality of care, optimizing workflows, and making informed strategic decisions.

KPIs in Radiology: In radiology, KPIs are specific metrics that evaluate the effectiveness of radiological practices (KPI in radiology) [20]. These metrics include turnaround times for imaging results, accuracy of diagnoses, patient wait times, and compliance with clinical guidelines. KPIs help radiology departments monitor and enhance their performance by providing a clear picture of how well they are meeting established standards and goals.

*Key performance indicators (KPIs) are fundamental in radiology for driving comprehensive quality improvements across all aspects of the field. By systematically measuring and analyzing KPIs, radiology departments can enhance not only patient care but also operational efficiency, resource allocation, and adherence to safety protocols. This continuous focus on quality through KPIs ensures that radiological practices consistently meet high standards, fostering better clinical outcomes, optimized workflows, and overall excellence in healthcare delivery [21,22]*.

*Key Performance Indicators (KPIs) in Radiology:* KPIs have become essential tools in the continuous improvement in healthcare quality. In radiology, KPIs serve as quantitative metrics that assess the performance and impact of radiological practices [21,22]. They are crucial for measuring clinical outcomes, optimizing resource allocation, and enhancing patient satisfaction. 

The International Society for Strategic Studies in Radiology (IS3R) highlights the strategic importance of KPIs, particularly in evaluating the integration of emerging technologies like AI. There is significant interest in developing KPIs that specifically assess AI’s integration into radiology and using AI to construct and refine these KPIs.

*The Role of AI in KPI Development:* AI’s advanced capabilities in data analyses and pattern recognition allow radiology departments to create more sophisticated KPIs that provide deeper insights into clinical workflows, diagnostic accuracy, and patient outcomes. AI can identify key performance indicators that may not be immediately apparent, uncovering hidden patterns in vast amounts of data. Moreover, AI facilitates real-time tracking and adjustment of KPIs, providing immediate feedback and actionable insights, enabling radiology practices to adapt quickly to changing conditions and emerging challenges. This dynamic approach ensures that KPIs remain relevant and accurately reflect the performance and integration of AI technologies.

*Predictive Analytics and Strategic Decision-Making:* AI-driven KPIs enhance predictive analytics, allowing radiology departments to proactively identify areas for improvement and innovation. This continuous evolution of KPIs, supported by AI, ensures that they not only measure current performance but also guide strategic decision-making and future advancements in radiology. By embedding AI into the KPI development process, radiology practices can achieve higher levels of precision, adaptability, and foresight, leading to superior healthcare delivery and enhanced patient care [21].

Overall, the integration of advanced technologies and the strategic use of KPIs are driving significant advancements in radiology. This evolution is closely aligned with the goals of precision and personalized medicine, where tailored treatments, enhanced safety, and superior patient outcomes are paramount.

### 1.4. A Narrative Review of KPI Integration in Radiology: Research Necessities and Objectives

The integration of advanced technologies such as X-rays, MRI, ultrasound, and PET scans in diagnostic and interventional radiology significantly enhances patient care and treatment outcomes. These technologies not only provide detailed internal imaging crucial for accurate diagnoses but also support personalized and precision medicine by enabling tailored treatment plans and reducing side effects. The ability to detect diseases early and monitor treatment responses allows for continuous adaptation of therapies, improving clinical outcomes and patient quality of life. Additionally, the multidisciplinary nature of radiology ensures comprehensive patient care through minimally invasive procedures, minimizing risks and recovery times.

Given the rapid advancements in radiology and the growing emphasis on integrating new technologies, there is a critical need for a narrative review focusing on key performance indicators (KPIs). KPIs are essential for measuring and improving the quality of radiological practices, encompassing aspects such as diagnostic accuracy, operational efficiency, and patient satisfaction. As the field evolves with the incorporation of emerging technologies like artificial intelligence and advanced imaging methods, it becomes increasingly important to understand and refine KPIs. This review’s *general aim* is to systematically explore the role of KPIs in enhancing radiology quality, identify current trends and gaps, and provide actionable recommendations for future research and practice.

The *specific aims* of this narrative review are to

1. *Assess how KPIs contribute to improving diagnostic accuracy, operational efficiency, and patient satisfaction in radiology.*
*(Key question: How do KPIs specifically contribute to improvements in diagnostic accuracy, operational efficiency, and patient satisfaction in radiology?)*
2. *Examine the impact of emerging technologies, particularly AI, on the development and application of KPIs.*
*(Key question: What is the impact of emerging technologies, particularly AI, on the development and effectiveness of KPIs in radiology?)*
3. *Identify research opportunities and areas needing improvement related to KPIs in radiology.*
*(Key question: What are the key research opportunities and areas for improvement in the use of KPIs within radiology?)*
4. *Offer recommendations for optimizing KPIs to drive continuous quality improvement in radiological services.*
*(Key question: What strategies can be recommended to optimize KPIs for driving continuous quality improvement in radiology?)*

This review will provide valuable insights into how KPIs can be leveraged to advance radiology practices and ensure high standards of quality in this vital field of medicine.

## 2. Methods

This overview of scientific literature used a standardized checklist for narrative review reporting (ANDJ Narrative Checklist. Available online: Narrative Review Checklist, available online: ANDJ checklist [23] (refer to the Supplementary Materials for the checklist grid and further references). The search was based on targeted searches (a) on Pubmed, Scopus, and Google Scholar; (b) properly designed assessment criteria for the study inclusion; (c) an assessment process; (d) a bias management strategy.

Studies from journals and/or conferences had to be peer-reviewed to be included in the process of preselection described below.

### 2.1. Search Strategies

The search was performed both in the *[Title/abstract]* and in the *full text*.

The components of this overview were obtained by means of the combination of two groups of keywords also combined with AND/OR Boolean logic of a search:

*Radiology Keywords*:

- Radiology;
- Medical imaging;
- Diagnostic imaging;
- CT scan;
- MRI;
- X-ray;
- Ultrasound;
- Nuclear medicine;
- Radiologist;
- Imaging technology;
- Radiographer.

*KPI Keywords*:

- Key performance indicators;
- KPI metrics;
- Performance measurement;
- Healthcare metrics;
- Data analytics;
- Performance indicators;
- Quality metrics;
- Efficiency metrics;
- Outcome measurement;
- Performance evaluation.

These groups can help focus searches on either the broad context of radiology and radiography or the specific performance and quality metrics used to evaluate these services. Table 1 reports the key searches with the aim of searching.

### 2.2. Assessment Criteria for Study Inclusion

To ensure a rigorous and high-quality narrative review, each selected study was assessed based on the following criteria (*refer to Supplementary Materials S2 for further references*):

*Clarity of Rationale (N1):* This criterion evaluates whether the study clearly articulates the reason for its investigation. The rationale should define the research problem, highlight its significance, and explain why the study is necessary. A well-defined rationale provides context and justifies the research effort. For instance, studies should outline the gap in existing knowledge or practice that the research aims to address, and the relevance of the study to the broader field of radiology and key performance indicators (KPIs).

*Design Appropriateness (N2):* This criterion assesses whether the study’s design is suitable for answering the research question or hypothesis. The design should align with the objectives and scope of the study. Appropriate design includes selecting the right methodology, sample size, and data collection methods. For example, if the study aims to analyze trends in KPIs over time, a longitudinal design would be appropriate, whereas a cross-sectional design might be used for a snapshot of current practices.

*Methodological Clarity (N3):* Methodological Clarity refers to the extent to which the study’s methods are described in detail and are replicable. This includes the transparency of procedures for the data collection, analysis, and interpretation. The study should provide clear information on how data were gathered, the tools and techniques used, and how the analysis was conducted. This clarity ensures that the study can be reproduced or critiqued based on the methodology described.

*Result Presentation (N4):* This criterion evaluates how effectively the study presents its findings. Results should be clearly organized, accurately reported, and appropriately interpreted. The presentation should include relevant tables, figures, and statistical analyses that support the conclusions drawn. The clarity of Result Presentation allows readers to understand and evaluate the study’s outcomes and their implications for radiology and KPIs.

*Justification of Conclusions (N5):* This criterion assesses whether the study’s conclusions are supported by its results. The study should provide a logical link between the data presented and the conclusions drawn. It should discuss the implications of the findings, address limitations, and suggest areas for future research. The Justification of Conclusions ensures that the study’s outcomes are valid and that the conclusions are based on sound evidence.

*Disclosure of Conflicts of Interest (N6):* Disclosure of Conflicts of Interest is crucial for assessing the impartiality and credibility of the study. This criterion checks whether the authors have declared any financial, professional, or personal interests that could bias the research. Full disclosure helps in evaluating the objectivity of the study and ensures that the findings are not influenced by external pressures or biases.

The choice of the component elements of this overview was made taking into account the 5 parameters (N1–N5) evaluated with a score from 1 = minimum to 5 = maximum and one parameter (N6) with a binary assessment (Yes/No). These parameters have been identified into the following: 

All the selected studies had to have the parameter N6 with “Yes” and the parameters N1-N5 with a score >3.

### 2.3. Assessment Process

Each study was reviewed by two assessors selected from the group comprising [DG], [AL], [YW], and [GL]. These assessors were tasked with evaluating each study based on *the focus on KPIs* and *after with the defined criteria*. Each criterion, including Clarity of Rationale, Design Appropriateness, Methodological Clarity, Result Presentation, Justification of Conclusions, and Disclosure of Conflicts of Interest, was scored on a predefined scale to provide a quantitative measure of each study’s quality and relevance.

The *primary assessors* independently reviewed the studies and assigned scores to each parameter, ensuring that each study was evaluated against the same standards. This dual-assessment approach was designed to enhance the reliability of this review by capturing different perspectives and reducing the likelihood of individual bias influencing the evaluation process.

In instances where the two initial assessors disagreed on the scores or the inclusion of a study, a third assessor from the group of [RR], [LL], [RAG], or [GM] was brought in to adjudicate. This third-party assessment was critical for resolving conflicts and ensuring that the final decisions were fair and well justified. The involvement of a third assessor helped to balance differing opinions and provided an additional layer of scrutiny to uphold the integrity of the review process.

The multi-assessor approach was implemented to minimize bias and ensure a thorough and balanced evaluation of the literature. By incorporating diverse viewpoints and providing a structured mechanism for resolving disagreements, this review aimed to offer a comprehensive and objective assessment of the studies related to key performance indicators (KPIs) in radiology.

### 2.4. Managing Bias in This Narrative Review

To ensure that this narrative review was objective and rigorous, several strategies were employed to manage and minimize bias throughout the assessment process. Here is how biases were managed:

*Diverse Assessors*:

Each study was reviewed by two primary assessors selected from the group comprising [DG], [AL], [YW], and [GL]. The inclusion of assessors from different backgrounds and expertise levels was intended to capture a range of perspectives and reduced the likelihood of individual biases influencing the evaluation process.

*Clear Assessment Criteria*:

The assessment was based on defined parameters such as Clarity of Rationale, Design Appropriateness, Methodological Clarity, Result Presentation, Justification of Conclusions, and Disclosure of Conflicts of Interest. Furthermore, data were presented based on a standardized checklist by using predefined parameters; the review process reduced the risk of subjective interpretation.

*Scoring System*:

Each parameter was scored on a scale from 1 to 5, with a binary assessment for the Disclosure of Conflicts of Interest (Yes/No). This quantifiable approach allowed for consistent evaluation across studies and provided a transparent mechanism for comparing study quality.

*Independent Review:*

The primary assessors independently reviewed the studies and assigned scores without consulting each other initially. This independence helped to ensure that individual judgments were based solely on the study’s merit and the predefined criteria, minimizing the influence of groupthink or shared biases.

*Dispute Resolution:*

In cases where the two primary assessors disagreed on scores or the inclusion of a study, a third assessor from the group of [RR], [LL], [RAG], or [GM] was involved to resolve the dispute. This third-party adjudication aimed to provide an impartial perspective and resolve conflicts fairly. The involvement of a third assessor added an extra layer of scrutiny and balance to the review process.

*Structured Mechanism for Disagreements:*

The process for resolving disagreements was structured and formalized. The third assessor reviewed the initial evaluations and provided a reasoned judgment to reconcile differences. This structured approach ensured that conflicts were addressed systematically and that final decisions were based on a comprehensive evaluation.

*Transparency:*

The use of a standardized checklist for presenting data and a clear scoring system provided transparency in the assessment process. By documenting the criteria and the scoring rationale, the review process was made transparent, allowing for a clear understanding of how decisions were made and reducing the potential for undisclosed biases.

By incorporating these strategies, this review aimed to offer a thorough and balanced evaluation of the literature on key performance indicators (KPIs) in radiology. The multi-assessor approach, coupled with structured criteria and formal dispute resolution, was designed to minimize bias and enhance the reliability and objectivity of the review process.

### 2.5. Selected Studies

**Figure 1:**
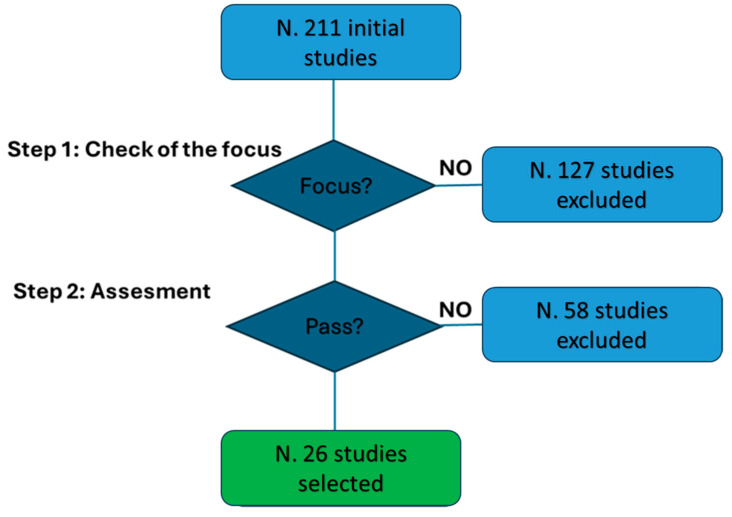
Details on the process of study selection.

The procedure ultimately identified 28 studies at the end of the selection process. Figure 1 outlines all the steps involved. This figure illustrates that the initial search yielded a total of 211 studies. From these, 127 studies were excluded due to their lack of focus on key performance indicators (KPIs). Following the evaluation according to the methodology described in Section 2.2 and Section 2.3, 26 studies were retained for further consideration, while 58 studies were excluded. See Table S1 for the narrative checklist.

## 3. Results

The results of this narrative review are organized into clear sections and subsections to provide a comprehensive understanding of the role of KPIs in radiology. This approach ensures that the key findings are presented logically and are easy to follow (section/subsections: focus and descriptions are introduced).

Section 3.1: Overview of Trends in Radiology KPIs

Section 3.1 offers an overview of the current trends in the use of key performance indicators (KPIs) within radiology, based on a focused search of the PubMed database. This section aims to give readers a clear picture of how KPIs are currently being used, what trends are emerging, and where there might be gaps or opportunities for further exploration.

- Section 3.1.1: Significance of Trend Analysis
  Section 3.1.1 emphasizes the importance of examining these trends. It discusses why understanding the trends in KPI usage is crucial for improving the quality of radiology services. This part highlights the need to track how KPIs are evolving and how these metrics are being applied to enhance diagnostic accuracy, efficiency, and patient satisfaction.
- Section 3.1.2: Analysis of Trends
  Section 3.1.2 delves into the trends themselves, analyzing the data collected from the PubMed searches. It looks at which KPIs are most commonly studied, how they are used, and what the findings suggest about the current state of the field. This section aims to paint a detailed picture of the landscape of KPI usage in radiology today.
- Section 3.1.3: Interpretation and Implications
  Section 3.1.3 interprets the trends identified in the previous section and discusses their implications for the future of radiology. This part explores how these trends might influence future practices, what challenges might arise, and how the use of KPIs might need to adapt in response to new technologies or changing patient needs.

Section 3.2: Outcomes of this Narrative Review

Section 3.2 presents the results of this narrative review in an organized format, broken down into specific subsections that correspond to this study’s aims.

- Section 3.2.1: General Findings from the Analysis
  Section 3.2.1 summarizes the general findings from this review, highlighting the key observations and categorizing them to provide a clear overview. This section gives a broad view of what this review uncovered about the current use of KPIs in radiology.
- Section 3.2.2: Addressing the Specific Aims
  Section 3.2.2 goes into detail on how this review addresses the four specific aims of this study, using key questions to guide the discussion.
  − Section 3.2.2.1 covers the first aim: Assessing how KPIs contribute to improving diagnostic accuracy, operational efficiency, and patient satisfaction in radiology.
    Key Question: How do KPIs specifically contribute to improvements in diagnostic accuracy, operational efficiency, and patient satisfaction in radiology?
  − Section 3.2.2.2 addresses the second aim: Examining the impact of emerging technologies, such as AI, on the development and application of KPIs.
    Key Question: What is the impact of emerging technologies, particularly AI, on the development and effectiveness of KPIs in radiology?
  − Section 3.2.2.3 discusses the third aim: Identifying research opportunities and areas needing improvement related to KPIs in radiology.
    Key Question: What are the key research opportunities and areas for improvement in the use of KPIs within radiology?
  − Section 3.2.2.4 focuses on the fourth aim: Offering recommendations for optimizing KPIs to drive continuous quality improvement in radiological services.
    Key Question: What strategies can be recommended to optimize KPIs for driving continuous quality improvement in radiology?

Section 3.3: Limitations and Considerations for Future Research

Section 3.3 discusses the limitations of this study and offers considerations for future research. This section acknowledges any constraints in the current review and suggests areas where future research could further expand on the findings or address any gaps identified.

Section 3.4: Synoptic diagram of the results

Section 3.4 reports a synoptic diagram providing a highly concise sketch of the results, organized into tabular connections and diagrams, aligned with the overall aim and specific objectives.

The results are supported by eight thematic tables, which help to organize and clarify the findings. Additionally, the Supplementary Materials includes detailed analytical summaries that focus on specific KPIs, providing even more in-depth information for those interested in the finer details.

### 3.1. The Trends in the Studies on KPIs in the Field of Radiology

#### 3.1.1. Why Analyzing Trends in PubMed Research on KPIs Is Crucial

Understanding trends in PubMed, one of the most important biomedical databases for research on KPIs in radiology and related fields, is crucial for several reasons:

*Tracking Research Progress:* Analyzing these trends helps identify how the focus of research has evolved over time. It provides insights into the progression of KPIs in radiology, reflecting shifts in research priorities and methodologies.

*Impact of External Factors:* The trends highlight how global events, such as the COVID-19 pandemic, significantly influence research activity. Observing these changes can offer valuable lessons on how external factors drive shifts in research focus and priorities.

*Identifying Research Gaps:* A low number of review articles, despite increased research activity, may suggest potential gaps in comprehensive evaluations of KPIs. Recognizing these gaps can guide future research efforts to ensure that all critical areas are addressed.

*Evaluating Technological Advances:* Trends in integrating AI with KPIs may reveal how technological advancements are applied to improve performance metrics. This analysis helps understand how AI contributes to optimizing KPIs and enhances decision-making processes.

#### 3.1.2. Trends in PubMed Overview

A search was conducted on the PubMed database with search criteria outlined in Box S2, and yielded a total of 23 studies on the application of KPIs in radiology or for radiographers since 2008.

Figure S4 illustrates the increase in the number of articles indexed in PubMed on the application and description of KPIs in radiology or dedicated for radiographers or radiologists, based on the search parameters given in Box S2.

Based on article types, a low prevalence of reviews (*n* = 5, 20.0%) concerning the application of VR in radiology emerged. 

Research on this topic has grown significantly during two key periods, as illustrated in Figure S4. The first major surge occurred in the past decade, from 2014 to the present, when 92.0% of all indexed articles on this subject appeared in the PubMed database. During this time, there was a notable increase in interest and collaborative efforts to use KPIs to improve and evaluate clinical outcomes in radiology and radiographer or radiologist clinical practice. This era saw a focused effort to use KPIs to enhance patient care quality, streamline radiological workflow, and ensure effective clinical practices.

The second wave of accelerated research began with the COVID-19 pandemic in 2020. From that year onwards, almost half of all articles on this topic (52.0%) were published. This wave was driven by the urgent need to optimize healthcare during an unprecedented crisis. Researchers and healthcare professionals quickly adapted KPIs to manage increased patient volumes, ensure the safety of healthcare professionals, and maintain the quality of radiological services during the pandemic. During this time, the crucial role of KPIs in enabling healthcare systems to respond quickly and effectively to new challenges became clear, emphasizing their importance for clinical practice and outcome measurement.

The concept of investigating AI using KPIs as well as applying AI to improve KPIs is crucial for enhancing performance and achieving strategic goals. KPIs provide measurable values that help in understanding how effectively AI technologies are being utilized, while AI can be leveraged to optimize these metrics, offering deeper insights and driving better decision-making. From the data analysis obtained by using the second search string, the integration of AI in various biomedical areas for KPIs has been a high priority over the past decade.

The numerical results and growing interest are quite similar for both search terms. Notably, of the 35 articles found in this final PubMed search, 88.5% were published in the past five years, i.e., after the COVID-19 pandemic. In this primary search, only 2 of the 35 articles deal with the application of KPIs, specifically in radiology, involving AI. 

#### 3.1.3. Emerging Implications 

Research on KPIs for radiology, radiographers, and radiologists has grown recently grown in the face of global healthcare challenges. The two rapid periods of research emphasize the dynamic and indispensable role of KPIs in radiology and radiographer practice. The findings and insights from these studies have advanced the field and demonstrated the critical importance of KPIs in maintaining and improving healthcare quality and efficiency during crises. While the integration of AI and KPIs in radiology is still in its infancy, there is already early evidence in the literature of evaluating radiology software with artificial intelligence in relation to KPIs.

*Advancements in KPI Research:* The increasing volume of studies on key performance indicators (KPIs) in radiology over the past decade underscores a significant shift towards data-driven practices. This trend highlights the growing recognition of KPIs as essential tools for improving clinical outcomes and optimizing radiological workflows. The surge in research from 2014 onward reflects a broader healthcare movement towards enhancing patient care and operational efficiency through measurable metrics.

*Impact of the COVID-19 Pandemic:* The COVID-19 pandemic has profoundly influenced research trends, as evidenced by the substantial rise in articles published post-2020. This accelerated focus on KPIs reveals their critical role in managing healthcare challenges during crises. The pandemic has demonstrated the necessity for adaptable and robust KPI frameworks to handle increased patient volumes and maintain care standards under unprecedented conditions.

*Research Gaps and Opportunities:* Despite the growing body of research, the low prevalence of review articles suggests notable gaps in the comprehensive assessment of KPI applications. This indicates a need for more integrative reviews that consolidate existing findings and offer a holistic view of KPI effectiveness and implementation in radiology. Addressing these gaps will be crucial for refining KPI practices and ensuring their optimal application in clinical settings.

*Integration of AI and KPIs:* The focus on integrating artificial intelligence (AI) with KPIs reflects ongoing efforts to enhance performance metrics through advanced technology. The intersection of AI and KPIs is poised to revolutionize healthcare practices by providing deeper insights and improving decision-making processes. However, while this integration is gaining momentum, further research is needed to fully harness AI’s potential in optimizing KPIs and addressing existing challenges.

Future Research Directions: The expansion of KPI research in radiology highlights the field’s progress and the emerging need for further exploration. Future research should prioritize filling identified gaps, such as the lack of comprehensive reviews and the deeper integration of AI with KPIs. This will help advance the field and contribute to more effective and efficient healthcare delivery.

In summary, the evolving research landscape on KPIs in radiology, driven by both technological advancements and global health challenges, emphasizes their growing importance in enhancing healthcare quality. The integration of AI into KPI frameworks holds promise for further advancements, though the field will benefit from continued research and more extensive evaluations to fully leverage these emerging technologies.

### 3.2. Outcome from the Analysis

#### 3.2.1. General Findings from the Analysis and Categorization 

KPIs serve as indispensable tools in contemporary healthcare management, facilitating the comprehensive evaluation, continuous monitoring, and targeted enhancement in operational efficiencies, care quality, and strategic decision-making (Harvey et al., 2023 [24]; Walther et al., 2023 [25]; Tanguay et al., 2023 [26]; Wihl et al., 2021 [27]; Fayemiwo et al., 2021 [28]). Across various healthcare domains, from clinical trials and diagnostic imaging to artificial intelligence (AI) integration and multidisciplinary team (MDT) collaborations in cancer care, KPIs provide actionable insights crucial for optimizing resource allocation and fostering continuous improvement (Teichgräber et al., 2021 [29]; Al Shawan, 2021 [30]; European Society of Radiology, 2020 [31]; Nason et al., 2020 [32]; Dick et al., 2021 [33]).

Harvey et al. (2023) [24] underscore the profound impact of cybersecurity incidents on clinical trial operations, revealing significant decreases in patient referrals and trial recruitment rates following a ransomware attack on the Irish health service. This event highlights vulnerabilities in healthcare IT infrastructure and emphasizes the critical need for robust cybersecurity measures to ensure uninterrupted patient care and clinical trial integrity.

Walther et al. (2023) [25] advocate for standardized KPIs in radiology to assess the appropriateness of diagnostic imaging, addressing the variability in methodologies across studies and the impact on clinical decision-making. By establishing unified measurement guidelines, healthcare providers can enhance diagnostic accuracy and treatment efficacy while maintaining high standards of care.

Tanguay et al. (2023) [26] propose frameworks for evaluating AI software in radiology, emphasizing the importance of standardized protocols to ensure patient safety and streamline integration into clinical workflows. These frameworks aim to optimize resource allocation and support evidence-based decision-making in healthcare settings.

Wihl et al. (2021) [27] highlight the pivotal role of KPIs in multidisciplinary team (MDT) meetings for cancer care, where radiology information significantly influences clinical decision-making processes. Effective use of KPIs in MDT settings enhances collaboration and improves patient outcomes by ensuring comprehensive case discussions and informed treatment strategies.

Fayemiwo et al. (2021) [28] demonstrate the effectiveness of Deep Transfer Learning models in enhancing radiological diagnostics during the COVID-19 pandemic, underscoring the role of KPIs in evaluating model accuracy and reliability. These advancements in AI-driven diagnostics contribute to improved patient care outcomes and diagnostic efficiency.

In alignment with strategic goals, Teichgräber et al. (2021) [29] introduce a Balanced Scorecard (BSC) tailored for radiology departments, integrating 18 daily and 10 annual KPIs to enhance operational efficiency and align departmental practices with stakeholder expectations. This approach ensures transparency, accountability, and continuous improvement in radiology service delivery.

Al Shawan (2021) [30] evaluates the impact of accreditation on radiology quality improvement at King Fahd Hospital, highlighting the role of KPIs in measuring and enhancing radiology reporting outcomes. Accreditation-driven quality initiatives underscore the importance of continuous performance monitoring and improvement in healthcare settings.

The European Society of Radiology (2020) [31] advocates for the implementation of performance indicators to enhance radiation protection practices across European radiology departments. These indicators support compliance with safety standards and facilitate continuous quality improvement through clinical audits and KPI-driven assessments.

Nason et al. (2020) [32] emphasize the role of KPIs in standardizing imaging protocols and supporting multidisciplinary team discussions in regionalized cancer care models. Effective use of KPIs ensures consistent quality of care and optimal patient management in specialized healthcare settings.

Dick et al. (2021) [33] survey global radiology quality improvement programs, highlighting KPIs as fundamental tools for standardizing practices and improving patient care outcomes. These initiatives promote evidence-based decision-making and enhance healthcare delivery across diverse international contexts. The European Society of Radiology (2020) [31] and subsequent studies by Nason et al. (2020) [32], Dick et al. (2021) [33], and Heilbrun et al. (2020) [34] explore the application of KPIs in enhancing radiation protection practices, improving cancer care through regionalization, and evaluating the impact of resident training on radiology department workflows. Raj et al. (2019) [35] and Pourmohammadi et al. (2018) [36] investigate KPIs in trauma care and hospital performance evaluations, respectively, highlighting their role in optimizing emergency response protocols and overall hospital efficiency. Obaro et al. (2018) [37] and Patel et al. (2017) [38] emphasize KPIs in evaluating screening methodologies and implementing quality improvement programs within radiology services. Rubin et al. (2017) [39], Karami and Safdari (2016) [40], and Schultz et al. (2016) [41] explore the use of KPIs in enhancing public education in radiology, developing performance dashboards for medical imaging departments, and improving radiation safety programs. Khalifa and Zabani (2016) [42], Harvey et al. (2016) [43], Karami (2016) [44], and Abujudeh et al. (2010) [45] further illustrate the comprehensive application of KPIs in monitoring ER performance, structuring quality assurance frameworks, designing radiology dashboards, and guiding organizational success in healthcare settings. Blakeley et al. [46] demonstrated through quantitative and qualitative methods that implementing radiographer-led image reading services in an emergency department led to notable improvements in key performance indicators (KPIs) such as image throughput, turnaround times, and diagnostic accuracy. KPIs are pivotal in enhancing various aspects of healthcare and education sectors, as particularly remarked in [47,48,49]. Whether applied in radiography for improving quality and safety, Koh et al. (2022) [47]; evaluating institutional effectiveness in allied healthcare education, Sreedharan et al. (2022) [48]; or optimizing workforce management, Lastrucci et al. (2024) [49], KPIs serve as essential tools for benchmarking, tracking performance, and driving continuous improvement. They facilitate objective assessment, enable targeted interventions, and ultimately contribute to elevating standards of service delivery and professional development within healthcare settings.

Collectively, these studies underscore the diverse applications and impacts of KPIs in healthcare, highlighting their essential role in driving continuous improvement, enhancing patient outcomes, and supporting evidence-based decision-making across various healthcare specialties.

Table 2 presents the key findings from the analysis of the 26 included studies.

Table 3 reports a categorization with the field/application of the KPIs.

#### 3.2.2. Detailed Answers to the Specific Aims

The specific contribution of this narrative review should be understood in the context of its defined aims because of the following: (a) Alignment with Objectives: This review’s findings are directly related to the goals set at the outset. Understanding these aims helps clarify how this review addresses specific research questions or issues. (b) Targeted Insights: This review offers insights tailored to its specific aims, making its conclusions and recommendations relevant to those areas. (c) Enhanced Understanding: Interpreting this review in light of its aims provides a clearer view of its impact and relevance in the field. (d) Guiding Future Research: This review’s aims help identify how its findings can shape future research directions and highlight any remaining gaps. (e) Understanding this review’s contributions alongside its specific aims ensures a complete appreciation of its value and role in advancing knowledge.

Thus, the contribution of this review is articulated in response to each specific aim.

##### 3.2.2.1. Answer to Specific Aim “Assess How KPIs Contribute to Improving Diagnostic Accuracy, Operational Efficiency, and Patient Satisfaction in Radiology”

Table 4 provides an in-depth analysis of the significant contributions various studies have made toward optimizing key performance indicators (KPIs) in radiological services. The focus is on three critical areas that are essential for the overall success and improvement in these services: diagnostic accuracy, operational efficiency, and patient satisfaction.

*Diagnostic Accuracy:* The table highlights how KPIs play a crucial role in refining diagnostic accuracy, a key element in radiology. Studies such as those by Fayemiwo et al. [28] and Blakeley et al. [46] illustrate the importance of precise imaging techniques and the correct application of diagnostic procedures. KPIs related to these areas ensure that radiological practices are both accurate and reliable, ultimately leading to better patient outcomes. Additionally, the implementation of effective quality assurance (QA) programs, as discussed in these studies, further supports the maintenance of high diagnostic standards, reducing errors, and improving overall patient care.

*Operational Efficiency:* Operational efficiency is another vital area where KPIs have a substantial impact. Studies like those by Tanguay et al. [26] and Karami [44] show that well-designed KPIs can lead to significant improvements in how radiological departments function. By optimizing resource allocation, streamlining processes, and integrating advanced technologies such as artificial intelligence (AI), these KPIs help enhance the overall efficiency of operations. This not only reduces costs but also accelerates the delivery of care, ensuring that patients receive timely and effective treatment. The focus on efficiency also contributes to better workload management and improved decision-making processes within radiology departments.

*Patient Satisfaction:* Patient satisfaction is a direct outcome of improvements in diagnostic accuracy and operational efficiency. Studies by Al Shawan [30] and Koh et al. [47] emphasize how KPIs can lead to higher levels of patient satisfaction by ensuring that services are both reliable and efficient. Enhanced diagnostic accuracy reduces the likelihood of misdiagnoses, which boosts patient confidence in the care they receive. Moreover, the efficient operation of radiological services means that patients experience shorter wait times and better overall service, contributing to higher satisfaction levels. KPIs that focus on patient outcomes and feedback are crucial for maintaining and improving the quality of care in radiological services.

Concluding this table underscores the comprehensive and multifaceted role that KPIs play in driving continuous quality improvement in radiological services. By focusing on diagnostic accuracy, operational efficiency, and patient satisfaction, these studies collectively provide valuable insights and recommendations for optimizing KPIs. The ultimate goal is to enhance the quality and effectiveness of radiological care, ensuring that it meets the evolving needs of patients and healthcare providers alike.

##### 3.2.2.2. Answer to Specific Aim “Examine the Impact of Emerging Technologies, Particularly AI, on the Development and Application of KPIs”

Table 5 highlights the transformative effect of emerging technologies on the development and application of key performance indicators (KPIs) in healthcare settings. Each entry underscores a specific technology’s role in enhancing KPI effectiveness, thus driving improvements in diagnostic accuracy, operational efficiency, and overall patient care.

Artificial intelligence (AI) plays a crucial role in advancing healthcare KPIs. Fayemiwo et al. [28] illustrate how Deep Transfer Learning frameworks, like VGG-16, can significantly boost diagnostic accuracy for COVID-19 classifications. KPIs such as the Matthews Correlation Coefficient (MCC) are employed to evaluate AI performance, demonstrating the technology’s impact on diagnostic precision. Tanguay et al. [26] propose a framework for evaluating AI software in radiology, emphasizing the need for standardized KPIs that ensure that AI integrates effectively into clinical workflows. This approach focuses on improving patient safety, clinical relevance, and operational efficiency.

Other emerging technologies also contribute significantly to refining KPIs. Nason et al. [32] discuss how telemedicine centralizes cancer care in Canada, improving KPIs related to patient access and timeliness by overcoming geographical and administrative barriers. .

Standardization and quality improvement in relation to high technology (HT) are vital for effective KPI application. Teichgräber et al. [29] introduce a Balanced Scorecard (BSC) tailored for radiology departments, which aligns strategic objectives with performance metrics. This approach enhances transparency and accountability through standardized KPIs. Al Shawan [30] evaluates the impact of Joint Commission International (JCI) accreditation on hospital quality, using KPIs to monitor improvements in patient outcomes and operational efficiency in relation to HT.

Performance measurement and management are critical for operational resilience. Harvey et al. [24] highlight the importance of resilient KPIs following a cyberattack on cancer trial operations, emphasizing the need for KPI monitoring to assess and enhance operational resilience. Walther et al. [25] advocate for uniform guidelines in diagnostic imaging KPIs to improve consistency and quality across radiology practices.

HT in radiation safety and education is also improved through KPIs. Shultz et al. [41] utilize KPIs to evaluate radiation safety programs, enhancing safety practices through continuous monitoring and data analyses. Patel et al. [38] detail a quality improvement program (QIP) in diagnostic imaging services, identifying measurable KPIs to foster continuous quality improvement.

Overall, emerging technologies, particularly AI, are revolutionizing KPI development and application in healthcare. These technologies enhance diagnostic accuracy, operational efficiency, and real-time performance monitoring, leading to more precise and actionable KPIs. Additionally technological advancements contribute to refining KPIs related to patient access, data security, and health monitoring. The integration of standardized frameworks and quality improvement initiatives further supports effective KPI use, ensuring enhanced performance and better patient outcomes across various healthcare settings.

##### 3.2.2.3. Answer to Specific Aim “Identify Research Opportunities and Areas Needing Improvement Related to KPIs in Radiology”

Recent studies across various healthcare domains underscore the critical role of key performance indicators (KPIs) not only in addressing current challenges but also in unlocking *significant opportunities for transformative improvements in healthcare delivery*.

Operational resilience and preparedness are critical, as highlighted by Harvey et al. [24], who underscored the vulnerability of clinical trial operations to cyber threats. This emphasizes the need/opportunity for healthcare systems to develop robust KPI frameworks and preparedness plans to mitigate risks, ensuring continuity in patient care and research activities.

Standardization in diagnostic imaging, as discussed by Walther et al. [25], reveals variability in criteria for assessing imaging appropriateness across modalities. Developing standardized KPI frameworks presents an opportunity to enhance decision-making consistency, optimize resource utilization, and ultimately improve patient outcomes through reliable clinical practices.

The integration of artificial intelligence (AI), proposed by Tanguay et al. [26], emphasizes the importance of standardized KPIs to ensure accurate performance assessment in radiology. This initiative offers opportunities to streamline AI adoption, enhance diagnostic accuracy, and improve operational efficiency within radiology practices.

KPIs such as completeness scores for radiology and patient-related information highlight opportunities to improve information integration and decision-making in MDT meetings [27]. Balancing contributions from diverse team members and enhancing leadership skills can optimize decision quality and patient outcomes in cancer care settings.

The KPIs in [28] involve accuracy metrics such as MCC and Kappa values, assessing the effectiveness of Deep Transfer Learning models (VGG-16 and VGG-19) in classifying COVID-19 from chest X-rays.

The implementation of Balanced Scorecards (BSCs), as introduced by Teichgräber et al. [29] for radiology departments, integrates 18 KPIs for daily monitoring. Such frameworks enhance transparency, accountability, and overall departmental performance across patient care, clinical outcomes, and operational productivity.

Quality improvement initiatives, exemplified by Al Shawan [30] evaluating JCI accreditation impacts, demonstrate significant improvements in various KPIs post-accreditation. This highlights opportunities for hospitals to leverage accreditation processes for enhancing patient outcomes, operational efficiency, and overall healthcare quality.

Radiation protection practices, developed by the European Society of Radiology [31], emphasize performance indicators to enhance safety in radiology departments. This approach optimizes safety protocols, improves patient and staff safety, and ensures compliance through continuous monitoring and KPI-driven insights.

Centralization and standardization efforts in cancer care, discussed by Nason et al. [32], highlight the role of KPIs in improving care outcomes. This presents an opportunity for healthcare systems to adopt centralized models supported by robust KPI frameworks, enhancing care quality, reducing costs, and improving patient access to specialized services.

Global variations in quality improvement programs and KPI implementations, noted by Dick et al. [33], underscore the need for standardized practices and international collaboration. Addressing these variations presents an opportunity to benchmark performance, drive continuous quality improvement, and optimize radiology services worldwide.

Efficiency in training programs, studied by Heilbrun et al. [34], uses TAT as a critical KPI to optimize radiology resident training impacts on patient care. This highlights opportunities for educational institutions and healthcare facilities to improve efficiency, reduce costs, and enhance patient care outcomes through targeted performance metrics.

Enhancing trauma care, evaluated by Raj et al. [35] using KPIs, identifies areas for improvement in trauma care protocols and patient outcomes. This offers opportunities to refine care delivery, optimize response times, and improve outcomes through continuous monitoring and KPI-driven improvements.

Public hospital performance, synthesized by Pourmohammadi et al. [36], emphasizes the importance of selecting appropriate KPIs to optimize hospital management and service delivery. This initiative offers opportunities for healthcare administrators to implement tailored KPI frameworks aligned with organizational goals, enhancing overall hospital performance.

Advancements in screening technologies, such as CT colonography discussed by Obaro et al. [37], leverage KPIs for optimizing screening pathways and improving population health outcomes. This presents opportunities to enhance screening efficacy, reduce costs, and improve health outcomes through evidence-based KPI monitoring.

Quality improvement programs in imaging services, detailed by Patel et al. [38], use measurable KPIs to foster a culture of continuous improvement and enhance service quality. This initiative offers opportunities for healthcare facilities to drive professional satisfaction, improve patient outcomes, and optimize service delivery through strategic quality metrics.

Public engagement in radiology education, analyzed by Rubin et al. [39], uses KPIs to measure the impact of public information portals on radiology education effectiveness. This presents opportunities for educational institutions and healthcare organizations to enhance public engagement, improve health literacy, and promote informed decision-making through strategic KPI-driven initiatives.

Dashboard development for medical imaging departments, pioneered by Karami and Safdari [40], uses KPIs to enhance operational transparency and decision-making. This presents opportunities for healthcare managers to implement data-driven strategies, optimize resource allocation, and improve departmental efficiency through comprehensive dashboard analytics.

Radiation safety programs, evaluated by Shultz et al. [41], use KPIs to enhance compliance and effectiveness in radiology departments. This initiative offers opportunities to strengthen safety protocols, improve staff training, and ensure regulatory compliance through continuous monitoring and KPI-driven evaluations.

Emergency room efficiency, assessed by Khalifa and Zabani [42] using KPIs, highlights opportunities to optimize patient flow, reduce wait times, and improve outcomes through streamlined processes and performance measurements.

Quality assurance in radiology operations, emphasized by Harvey et al. [43], uses KPI-driven frameworks to monitor and respond to quality issues. This offers opportunities for radiology departments to enhance service quality, improve stakeholder confidence, and achieve better patient care outcomes through systematic performance evaluations.

Dashboard design for performance optimization in radiology departments, outlined by Karami [44], focuses on relevant KPIs for enhancing departmental performance and decision-making. This initiative presents opportunities for healthcare administrators to improve data visualization, empower decision-makers, and drive continuous improvement through user-friendly dashboards.

Quality initiatives for radiology departments, discussed by Abujudeh [45], propose tailored KPIs to measure performance and support strategic goals. This offers opportunities for healthcare leaders to foster excellence, drive continuous improvement, and enhance patient care outcomes through targeted quality metrics.

Radiographer-led services in emergency departments, evaluated by Blakeley et al. [46], show improvements in efficiency and patient care. This initiative presents opportunities to optimize teamwork, enhance service delivery, and improve diagnostic accuracy through innovative service models supported by KPI-driven evaluations.

Performance monitoring in radiography, analyzed by Koh et al. [47], focuses on quality improvements and safety measures using KPIs. This presents opportunities for radiographers and healthcare managers to optimize workflow efficiency, reduce errors, and improve patient outcomes through targeted performance measurements.

Educational performance in allied healthcare, explored by Sreedharan et al. [48], emphasizes institutional KPI frameworks to assess teaching effectiveness. This offers opportunities for educational institutions to enhance teaching quality, improve student outcomes, and promote continuous educational improvement through comprehensive performance metrics.

Work shift optimization tools like the Skills’ Retention Monitoring (SRH) tool, introduced by Lastrucci et al. [49], use KPIs to track competencies and improve service delivery. This initiative presents opportunities for healthcare organizations to enhance workforce management, reduce costs, and elevate patient care through data-driven insights and continuous performance monitoring.

Overall, the integration of robust KPI frameworks across healthcare domains presents significant opportunities to enhance operational efficiency, improve clinical outcomes, optimize resource utilization, and drive continuous quality improvement. Table 6 reports a sketch of the opportunities.

While existing research has made significant strides in implementing KPI frameworks across different healthcare domains, several areas also emerge that warrant further exploration and research to advance the field.

Firstly, in the realm of cybersecurity and resilience in clinical trial operations highlighted by Harvey et al. [24], further investigation could focus on developing more sophisticated KPIs that not only measure preparedness but also assess the effectiveness of response strategies to cyber threats in real-time scenarios.

Secondly, the standardization of KPI frameworks in diagnostic imaging, as discussed by Walther et al. [25], presents an opportunity for future research to delve deeper into the development of adaptive KPI metrics that can accommodate technological advancements in imaging modalities and evolving clinical needs.

The integration of artificial intelligence (AI) in radiology, as proposed by Tanguay et al. [26], suggests avenues for research into refining KPIs specifically tailored to assess the ethical implications, patient outcomes, and long-term impacts of AI technologies in clinical practice.

Further exploration into the effectiveness of multidisciplinary team (MDT) meetings in cancer care, as studied by Wihl et al. [27], could involve developing comprehensive KPIs that measure the collaborative decision-making process across diverse healthcare professionals and its direct correlation with patient outcomes.

In the realm of deep learning for COVID-19 diagnoses using chest X-ray images, Fayemiwo et al. [28] showed promising results with existing KPIs. Future research could explore expanding these KPIs to encompass broader diagnostic capabilities across various respiratory illnesses and explore their application in real-world clinical settings.

Balanced Scorecards in radiology departments, advocated for by Teichgräber et al. [29], suggest opportunities for further research into developing dynamic KPIs that not only monitor daily operations but also facilitate predictive analytics to optimize resource allocation and enhance patient care pathways.

Quality improvement initiatives in radiology, such as those discussed by Dick et al. [33], highlight the need for research into standardized global benchmarks and KPIs that can facilitate cross-border comparisons and drive continuous quality improvement efforts.

Efficiency in radiology training programs, as examined by Heilbrun et al. [34], could benefit from further research into KPIs that measure educational outcomes, competency acquisition, and their direct impact on patient care quality and safety.

Raj et al. [35] evaluated trauma care system performance using KPIs such as response times and diagnostic turnaround. Further research could explore the development of more nuanced KPIs that encompass patient outcomes beyond initial response metrics, including long-term recovery and quality of life measures.

Pourmohammadi et al. [36] synthesized evidence on performance indicators for public hospitals, emphasizing the need for tailored KPIs that align with efficiency, effectiveness, and financial aspects. Future studies could focus on refining these indicators to capture more granular data on resource utilization and patient-centered outcomes.

Obaro et al. [37] discussed advancements in CT colonography for colorectal cancer screening, underscoring the role of KPIs in assessing diagnostic accuracy and cost-effectiveness. Further research might explore the integration of novel KPIs to evaluate the broader impact of screening programs on population health outcomes and healthcare resource allocation.

Patel et al. [38] detailed the implementation of quality improvement programs (QIPs) in imaging services, highlighting KPIs across safety, process improvement, and professional outcomes. Future studies could investigate the scalability of these programs across different healthcare settings and their long-term sustainability in driving continuous quality improvement.

Rubin et al. [39] examined the impact of public information portals on radiology education, using KPIs to measure effectiveness in enhancing patient engagement and health literacy. Further research could explore innovative uses of KPIs to optimize content delivery and personalize educational resources for diverse patient populations.

Karami and Safdari [40] developed performance dashboards for medical imaging departments, leveraging numerous KPIs to enhance operational transparency and decision-making. Future studies might focus on refining dashboard functionalities to integrate real-time data analytics and predictive modeling for proactive healthcare management.

Shultz et al. [41] evaluated radiation safety programs using KPIs to monitor equipment usage, staff training, and compliance with safety protocols. Further research could explore the development of standardized KPIs to benchmark radiation safety practices globally and drive continuous improvement in patient and staff safety.

Khalifa and Zabani [42] established KPIs for monitoring and improving emergency room (ER) performance, emphasizing patient flow and operational efficiency. Future studies could investigate the impact of telemedicine and digital health technologies on ER KPIs, aiming to optimize resource allocation and enhance patient care outcomes.

Harvey et al. [43] emphasized KPI-driven quality assurance in radiology operations, advocating for structured frameworks to monitor and respond to quality issues. Further research could explore the integration of artificial intelligence and machine learning algorithms to automate QA processes and enhance diagnostic accuracy.

Karami [44] introduced a design protocol for radiology dashboards, focusing on KPIs relevant to departmental performance. Future studies might investigate the usability and effectiveness of these dashboards in supporting clinical decision-making and improving workflow efficiency in radiology departments.

Abujudeh [45] discussed the role of KPIs in quality initiatives for radiology departments, proposing tailored indicators to measure performance and support strategic goals. Further research could explore the development of benchmarking tools and international collaborations to standardize KPIs and drive global quality improvement efforts.

Blakeley et al. [46] evaluated radiographer-led image reading services in emergency departments, highlighting improvements in efficiency and patient care. Future studies could investigate the scalability of these services and explore KPIs that measure interdisciplinary collaboration and their impact on diagnostic accuracy.

Koh et al. [47] used KPIs to monitor and enhance radiography performance, focusing on quality improvements and safety measures. Further research might explore the application of KPIs in emerging technologies such as artificial intelligence to optimize workflow efficiency and improve patient outcomes.

Sreedharan et al. [48] addressed performance evaluation in allied healthcare education, advocating for institutional KPI frameworks to assess effectiveness. Future studies could focus on developing KPIs that measure competency acquisition and the impact of educational interventions on patient care outcomes.

Lastrucci et al. [49] introduced the Skills’ Retention Monitoring (SRH) tool for optimizing work shifts in healthcare, using KPIs to track competencies and enhance service delivery. Further research could explore the integration of SRH with predictive analytics to optimize workforce management and improve patient care quality.

Table 7 reports a sketch with the suggestions for further research.

##### 3.2.2.4. Answer to Specific Aim “Offer Recommendations for Optimizing KPIs to Drive Continuous Quality Improvement in Radiological Services”

Table 8 presents the recommendations identified for optimizing KPIs to drive continuous quality improvement in radiological services. The following eight key themes were identified:

*Standardization of KPIs:* Developing and implementing standardized KPIs are fundamental to achieving consistency and reliability in diagnostic practices. As highlighted by Walther et al. [25], Tanguay et al. [26], and Teichgräber et al. [29], uniform guidelines help establish clear benchmarks, making it easier to track and compare performance across different institutions.

*Integration of Advanced Technologies:* The integration of technologies such as AI as discussed by Fayemiwo et al. [28] could be useful in perspective. This technological advancement allows for more accurate data collection and better tracking of performance metrics.

*Utilization of Performance Dashboards:* Performance dashboards offer a powerful tool for visualizing KPIs, as demonstrated by Karami and Safdari [40] and Karami [44]. They enable real-time insights into performance, allowing for a quick identification of trends and decision-making based on comprehensive data.

*Adoption of Quality Improvement Programs (QIPs):* Implementing QIPs is crucial for continuous improvement. Patel et al. [38] and Pourmohammadi et al. [36] emphasize that regular assessment and refinement in KPIs through QIPs ensure that performance issues are systematically addressed, leading to enhanced service quality.

*Focus on Patient-Centered Metrics:* Aligning KPIs with patient outcomes and satisfaction is essential. As noted by Nason et al. [32] and Heilbrun et al. [34], prioritizing metrics that directly affect patient care ensures that quality improvement efforts are focused on meeting patient needs and enhancing their overall experience.

*Establishment of Monitoring and Feedback Mechanisms:* Continuous monitoring and feedback are vital for driving improvements. Shultz et al. [41] and Raj et al. [35] highlight the importance of these mechanisms in identifying and addressing performance issues early, thereby facilitating ongoing enhancements.

*Enhancement in Education and Training:* Regularly assessing educational tools and ensuring that staff are well trained impact KPI effectiveness. Rubin et al. [39] suggest that effective training and updated knowledge are crucial for proper KPI implementation and overall service quality.

*Emphasis on Safety and Quality Assurance:* Maintaining high safety and quality standards is critical for building patient trust and satisfaction. As outlined by the European Society of Radiology [31] and Blakeley et al. [46], focusing on these areas ensures that KPIs reflect best practices and contribute to continuous quality improvement.

### 3.3. Limitations and Considerations for Future Research

While the studies reviewed offer valuable insights into optimizing KPIs for radiological services, there are a few considerations to keep in mind:

*Diverse Contexts and Methodologies:*

Varied Settings: The research includes studies conducted across a range of institutions and settings, such as those by Tanguay et al. [26] and Karami and Safdari [40]. This diversity enriches the findings but also means that results may be more context-specific. Future research could benefit from exploring how these insights apply across different types of healthcare environments, including smaller or rural facilities.

Methodological Differences: Studies like those by Patel et al. [38] and Rubin et al. [39] use various methodologies to assess KPIs. While this variety demonstrates the broad applicability of KPI concepts, it also highlights the need for more standardized approaches to ensure consistency in results. Continued efforts to harmonize KPI measurement techniques could enhance the comparability of findings.

*Potential Biases:*

Publication Trends: The studies reviewed, such as Walther et al. [25] and Teichgräber et al. [29], may reflect trends in published research. Positive results are often more likely to be published, which can provide an optimistic view of KPI effectiveness. A balanced approach that includes both successful and less successful implementations could offer a more comprehensive understanding of KPI impact.

Focus on Specific Outcomes: Some studies, including those by Fayemiwo et al. [28] and Shultz et al. [41], focus on particular aspects of KPI performance. While this targeted approach provides depth, integrating findings from a broader range of outcomes can offer a more holistic view of KPI effectiveness.

*Generalizability and Application:*

Context-Specific Findings: Research conducted in high-tech or urban settings, such as that by Nason et al. [32], offers valuable insights but may be most directly applicable to similar environments. Future studies could explore how these findings translate to different settings, including those with varying levels of technological resources or patient demographics.

Evolution of Practices: The rapid advancement in radiology practices and technologies, as noted in studies like those by Harvey et al. [24] and Wihl et al. [27], means that some findings might evolve over time. Ongoing research is essential to ensure that KPI recommendations remain relevant and incorporate the latest advancements.

*Data Quality and Measurement Tools:*

Accuracy and Consistency: Ensuring the accuracy of data collection and reporting, as emphasized by Karami [44] and Khalifa and Zabani [42], is crucial for the effectiveness of KPIs. Continued efforts to refine measurement tools and data reporting practices will enhance the reliability of KPI assessments and their applicability across different settings.

Overall, the studies reviewed offer a strong foundation for optimizing KPIs in radiological services. Addressing these considerations can further enhance the application and impact of KPIs, ensuring continuous improvement and better alignment with diverse healthcare needs.

Table 9 reports a sketch on the limitations and recommendations for enhancing the generalizability of KPI studies in radiological services.

### 3.4. Synoptic Diagram of Results

**Figure 2:**
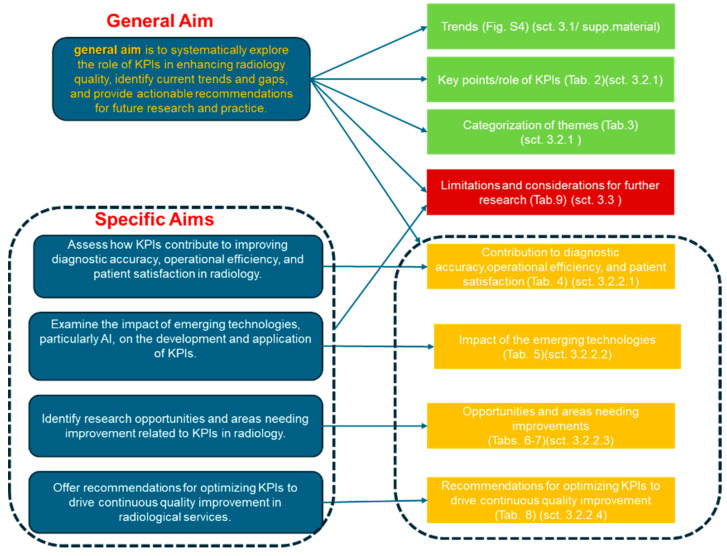
Synoptic diagram for tabular reporting of results.

The diagram in Figure 2 provides a highly concise sketch of the results, organized into tabular connections and diagrams, aligned with the overall aim and specific objectives.

**Block 1** (from top to bottom) highlights trends that are further supported by the diagram in the Supplementary Materials (Figure S4). **Block 2** references Table 2, which emphasizes the key points and the specific role of KPIs in each analyzed study. **Block 3** focuses on the categorization of emerging trends as shown in Table 3.

In direct connection with the specific aims and aligned with the general objective, we find the following:

**Block 5**, which addresses the contribution of KPIs in terms of diagnostic accuracy, operational efficiency, and patient satisfaction, as presented in Table 4.

**Block 6**, which explores the role of KPIs in relation to emerging technologies (including AI), as detailed in Table 5.

**Block 7**, which references Table 6; Table 7, dedicated to illustrating both the opportunities and areas in need of further research.

Finally, **Block 8** summarizes the recommendations from the studies to optimize KPIs for driving continuous quality improvement in radiology, as presented in Table 8. Block 4 (highlighted in red) points out the potential contextual limitations, biases, and generalizability concerns of the analyzed studies, as reported in Table 9.

## 4. Discussion

The discussion on KPI integration in radiology is organized into several sections and subsections to comprehensively address various aspects.

*Section 4.1* delves into the key roles of KPIs in radiology, examining the following:

- *The Evolution of Imaging Technologies*: This includes the transition from traditional X-ray radiography to advanced modalities such as CT scans, MRIs, PET-CT, and PET-MRI. KPIs are crucial for assessing the performance and quality of these diverse imaging techniques.
- *Radiological Safety:* Emphasizes minimizing radiation exposure for patients and healthcare providers through stringent protocols, quality assurance programs, and regular safety audits. KPIs measure adherence to these safety standards.
- *Integration of New Technologies:* Focuses on the incorporation of innovative technologies like artificial intelligence (AI), augmented reality (AR), and virtual reality (VR). KPIs are essential for evaluating their impact on diagnostic accuracy, workflow efficiency, and user satisfaction.
- *Telehealth and Digital Health:* Covers the adoption of network technologies (but not as additions) for secure image sharing and the expansion of tele-radiology. KPIs evaluate the effectiveness of these digital health technologies in improving access to care and diagnostic efficiency.
- *Roles of Radiologists and Radiographers:* Highlights the importance of KPIs in optimizing the performance of radiologists and radiographers, focusing on diagnostic accuracy, image quality, patient satisfaction, and procedural efficiency.
- *Strategic Initiatives by Associations:* Examines how national and international scientific societies promote evidence-based practices, set training standards, and advocate for policy improvements. KPIs provide insights into the effectiveness of these initiatives.

*Section 4.2* addresses the importance and limitations of the scientific literature on KPIs in radiology. It outlines recommendations for the more effective and detailed integration of KPIs into healthcare systems. The section underscores the need for active involvement from national and international associations in developing and implementing these KPIs to ensure their successful integration.

*Section 4.3* builds upon the limitations and recommendations discussed earlier, providing an in-depth analysis from three distinct perspectives, detailed in the following subsections:

- *Section 4.3.1:* Analyzes documents from scientific associations focused on digital health and telehealth, including applications relevant to radiology such as tele-radiology. This analysis helps understand how these technologies can be integrated into radiological practices and assessed through appropriate KPIs.
- *Section 4.3.2:* Reviews documents from scientific associations that concentrate on radiology and radiation protection. This subsection explores how KPIs can be applied to enhance radiological safety and quality assurance practices.
- *Section 4.3.3:* Outlines future development pathways where KPI definitions will be critical. This includes not only the ongoing integration of AI and advancements in health technology but also new developments in CAD/CAM integration, tele-radiology, and home-based radiology. The section also emphasizes the increasing importance of patient-centered care and economic factors in KPI development.

*Section 4.4* reports a concise synoptic diagram providing a highly concise sketch of the discussion, organized into tabular connections and diagrams, aligned with the overall development of the discourse.

*Section 4.5* addresses the limitations of this study, acknowledging the constraints inherent to the narrative review methodology. It highlights the specific limitations of this approach and discusses how this review was complemented by available international documents accessible online. Due to logistical constraints and the scope of this overview study, it was not feasible to include non-digitized documents within the timeframe. Consequently, this study relied on online resources to provide a comprehensive analysis.

### 4.1. KPIs and the Key Areas of Radiology: From Technological Advances to Safety Protocols

Radiology has a long and significant history, encompassing both diagnostic imaging—now integrated with interventional solutions—and radiotherapy, which provides imaging for treatment planning. Since the 1950s, there has been a growing focus on quality control within this field [18], and key performance indicators (KPIs) have become crucial tools in assessing and improving practices.

*When discussing the outcomes of this review, it is important to highlight several key aspects where KPIs play a critical role, which emerged from this overview:*

*Imaging Practice Evolution:* Radiology’s evolution from X-ray radiography to include CT scans, ultrasound, MRI, and hybrid methods like PET-CT and PET-MRI reflects significant advancements in imaging technology [51,52]. KPIs are essential for tracking the performance and quality of these diverse modalities. Key indicators include diagnostic accuracy, image quality, and efficiency of imaging processes. By monitoring these KPIs, we ensure that advancements in imaging technology continue to meet high standards and contribute effectively to clinical practice.

*Radiological Safety:* The emphasis on radiological safety involves minimizing radiation exposure to patients and healthcare providers [31]. This includes stringent protocols, quality assurance programs, equipment calibration, and regular safety audits. KPIs in radiological safety measure adherence to safety standards, including radiation dose levels, audit results, and the effectiveness of protective measures. These indicators are vital for ensuring that safety protocols are rigorously followed and continuously improved.

*Integration of Innovative Technologies:* The integration of augmented reality (AR), virtual reality (VR), and artificial intelligence (AI) [52] is transforming radiology. AR and VR enhance visualization for surgical planning and education, while AI improves image analyses and clinical decision-making. KPIs are crucial for evaluating the impact of these technologies. Metrics such as improvements in diagnostic accuracy, workflow efficiency, and user satisfaction help assess how well these technologies are integrated into clinical practice and their effectiveness in enhancing patient care.

*Role of Artificial Intelligence (AI):* AI is emerging as a transformative (and distinct from the other emerging technologies) force in radiology, assisting with image interpretation, diagnoses, and performance evaluation [53]. KPIs track the effectiveness of AI systems by measuring diagnostic accuracy, turnaround times, and resource optimization. These indicators help evaluate AI’s contribution to improving diagnostic capabilities and operational efficiency, guiding its integration into healthcare practices.

*Digital Health and Tele-radiology:* The adoption of network technologies for secure image sharing and the growth of tele-radiology [54] highlights the shift towards digital health. KPIs measure the effectiveness of these digital health technologies through metrics such as image transfer times, diagnostic report turnaround, and consultation success rates. These indicators are essential for assessing how well digital health initiatives improve patient outcomes and enhance healthcare delivery [55].

*Roles of Radiologists and Radiographers:* Radiologists and radiographers are pivotal in interpreting images, diagnosing conditions, and ensuring patient safety [4]. KPIs optimize their performance by tracking metrics such as diagnostic accuracy, image quality, and patient satisfaction. Continuous professional development and performance metrics are crucial for advancing the field and improving patient care.

*Strategic Initiatives by Associations:* National and international scientific societies play a key role in shaping the future of radiology by promoting evidence-based practices and setting training standards [31]. KPIs provide insights into the effectiveness of these initiatives, including adherence to guidelines, research advancements, and policy impacts. These indicators help understand current trends and guide future directions in radiology.

Overall, KPIs are integral to monitoring and enhancing various aspects of radiology, from technological advancements and safety to performance and strategic initiatives. They ensure continuous improvement and high standards in patient care and clinical practice.

Table 10 reports a sketch of these considerations.

### 4.2. Emerging Insights for Successful KPI Integration and Utilization

This review of the scientific literature has highlighted both promising opportunities and areas that require further investigation. While the literature extensively addresses the specific applications of KPIs, it reveals a noticeable gap in their integration into stable and routine healthcare systems. It is important to recognize that the role of scholars is primarily to develop, test, and research these tools rather than to implement them directly into healthcare systems. Scholars are tasked with the following:

- *Developing KPIs:* Creating and refining KPI frameworks based on theoretical and empirical data.
- *Testing and Validating:* Evaluating the effectiveness and reliability of KPIs through rigorous research.
- *Conducting Research:* Exploring new methodologies and technologies to improve KPIs.
- *Publishing Findings:* Disseminating research to advance understanding and inform practice.

However, the integration and standardization of KPIs into healthcare systems require additional efforts. This involves the following:

- *Collaboration with Healthcare Providers:* Ensuring that KPIs are adapted to fit clinical workflows and patient care practices.
- *Development of Implementation Strategies*: Creating frameworks for the practical application of KPIs, including training and infrastructure adjustments.
- *National and International Standardization:* Aligning KPI standards across regions to ensure consistency and reliability, requiring coordination with national and international bodies.
- *Continuous Monitoring and Adjustment:* Regularly evaluating and refining KPIs based on real-world feedback and performance data.

The effective integration of KPIs into healthcare systems demands a concerted effort from researchers, healthcare professionals, and policymakers to bridge the gap between research and practical application.

*The seven aspects highlighted in Table 10, where KPIs play a critical role, therefore require systematic and effective attention through national and international initiatives.*

*The authors have provided significant recommendations for advancing these tools and directing future efforts. These recommendations emphasize the need for coordinated actions at a broader scale:*

- *Standardization of KPIs:* Developing and implementing standardized KPIs are essential for achieving consistency and reliability in diagnostic practices. Uniform guidelines help establish clear benchmarks, making it easier to track and compare performance across institutions globally. Walther et al. [25], Tanguay et al. [26], and Teichgräber et al. [29] advocate for this approach, which necessitates national and international collaboration to create and enforce these standards. National radiological societies and international organizations, such as the Radiological Society of North America (RSNA) and the European Society of Radiology (ESR), should spearhead these efforts to ensure widespread adoption and adherence.
- *Integration of Advanced Technologies:* The integration of advanced technologies, including artificial intelligence (AI) enhances KPI precision and provides real-time monitoring capabilities. This integration facilitates more accurate data collection and performance tracking. Fayemiwo et al. [28] highlight the importance of incorporating these technologies into KPI frameworks. National health organizations and international bodies should support initiatives that promote the integration of these technologies through grants, research collaborations, and shared technological platforms.
- *Utilization of Performance Dashboards:* Performance dashboards are instrumental in visualizing KPIs, offering real-time insights, and facilitating data-driven decision-making. Karami and Safdari [40] and Karami [44] demonstrate the effectiveness of these tools. The development and adoption of standardized performance dashboards could be promoted through international consortia and national radiology associations, which can provide guidelines and best practices for implementing these tools across various healthcare settings.
- *Adoption of Quality Improvement Programs (QIPs):* Quality improvement programs (QIPs) are vital for the continuous enhancement in radiological services. Patel et al. [38] and Pourmohammadi et al. [36] emphasize the need for regular assessment and refinement in KPIs through QIPs. National and international radiology organizations should advocate for the establishment of QIPs, provide training resources, and facilitate knowledge exchange to ensure effective implementation and continuous quality improvement.
- *Focus on Patient-Centered Metrics:* Aligning KPIs with patient outcomes and satisfaction is crucial for ensuring that quality improvement efforts are patient-focused. Nason et al. [32] and Heilbrun et al. [34] highlight the importance of this alignment. National health agencies and international organizations should lead initiatives that promote the development of patient-centered KPIs, ensuring that these metrics are integrated into clinical practice guidelines and quality assessment frameworks.
- *Establishment of Monitoring and Feedback Mechanisms:* Continuous monitoring and feedback are essential for identifying and addressing performance issues. Shultz et al. [41] and Raj et al. [35] underscore the significance of these mechanisms. National and international radiological societies should develop and support systems for ongoing monitoring and feedback, facilitating the early identification of performance issues and driving improvements across the field.
- *Enhancement in Education and Training:* Effective education and training impact the successful implementation of KPIs. Rubin et al. [39] emphasize the importance of up-to-date training and knowledge. National radiology boards and international educational organizations should prioritize the development of comprehensive training programs and resources, ensuring that radiologists and radiographers are equipped with the skills needed to implement and utilize KPIs effectively.
- *Emphasis on Safety and Quality Assurance:* Maintaining high safety and quality standards is essential for building patient trust and improving care. The European Society of Radiology [31] and Blakeley et al. [46] stress the need for a strong focus on safety and quality assurance. National and international initiatives should include rigorous safety and quality assurance programs, promoting adherence to best practices and ensuring continuous improvement in radiological services.

Additionally, the evolving landscape of radiology presents new challenges and opportunities. The integration of CAD-CAM technology for radiotherapy masks poses significant implementation and standardization challenges. Similarly, home-based radiology and tele-radiology introduce new demands that KPIs must address effectively. As remote diagnostics and the miniaturization of technologies advance, national and international radiological communities must collaboratively develop and refine KPI frameworks to accommodate these innovations.

Overall, addressing these evolving scenarios and integrating new challenges into KPI frameworks will require coordinated efforts at both national and international levels. By fostering collaboration among radiology societies, health organizations, and technological developers, the field can advance in a manner that enhances patient care and ensures continuous improvement in radiological practices.

We can summarize the findings presented in Table 11 as follows:

**Notes:**

- Standardization and Integration: Ensuring KPIs are standardized and integrated requires coordinated actions from both national and international organizations to provide a cohesive framework for application.
- Advanced Technologies: Emphasizing the role of advanced technologies like AI can enhance the effectiveness of KPIs, but requires collaborative support and resources.
- Patient-Centered Focus: Aligning KPIs with patient outcomes ensures that improvements are directly beneficial to patient care.
- Continuous Improvement: Effective monitoring, feedback, education, and safety measures are crucial for the ongoing enhancement and successful integration of KPIs into healthcare systems.

### 4.3. A Guard to International Documents Provided by National and International Bodies

It is interesting based on the recommendations in Table 11 to complement the discussion by examining the stance of both national and international entities in this field and/or closely related areas (such as integration with digital health) and to point out the future expected directions of evolutions of radiology employment. For the practical feasibility of this complementation, we have considered entities that readily provide documents on KPIs online. Many other organizations actively address these issues but do not directly publish documents online. To locate such documents would require a three-level approach—contacting the entity’s contact person, requesting the document, and obtaining it—which is time-consuming and impractical for a review paper.

#### 4.3.1. Focus on Digital Health and Telehealth Integration

Regarding integration with digital health and telehealth, it is useful to highlight the positions of some national and international entities. As highlighted in [55], the analysis of select entities and institutions reveals a comprehensive and varied perspective on telehealth practices and their evaluation [56,57,58,59,60], which is also applicable to integrating radiology into digital health and telehealth. These diverse focuses collectively contribute to a holistic understanding, influenced by the roles and functions of different institutions within the healthcare landscape.

A World Health Organization (WHO) document [56] underscores the importance of demonstrating telehealth benefits during healthcare service transitions. It proposes evaluation indicators categorized into short-term, medium-term, and long-term outcomes, focusing on metrics such as increased teleconsultations, patient savings, and remote patient monitoring. Conversely, an American Telemedicine Association (ATA) document [57] emphasizes balancing clinical excellence with operational efficiency in telehealth. It addresses challenges in delivering quality care remotely and suggests solutions like configurable clinical dashboards and real-time quality assurance reports. Similarly, the American College of Physicians (ACP) [58] recommends developing telehealth-specific KPIs based on quality measurement principles from in-person care. It advocates for reliable and valid performance measures tailored to telehealth environments while mitigating unintended consequences, particularly concerning disadvantaged communities. A National Health Service (NHS) document [59] outlines KPIs for Integrated Urgent Care (IUC), integrating telehealth to optimize healthcare delivery. It focuses on metrics like abandoned calls, call response times, and the proportion of calls assessed by a doctor within a specified timeframe. Lastly, the Dubai Health Authority guidelines [60] establish procedures for reporting telehealth KPIs to enhance patient quality and safety and promote healthcare sector growth. These guidelines categorize KPIs into access and quality metrics, covering areas such as patient waiting times, population coverage, medication prescriptions, and patient and staff satisfaction. These documents, also applicable to radiology, provide valuable insights into evaluating telehealth practices, highlighting the need to demonstrate benefits, balance clinical excellence with operational efficiency, apply quality measurement principles, integrate telehealth into healthcare frameworks, and ensure patient safety and satisfaction. Table 12 synthesizes the diverse KPIs provided by each analyzed entity.

#### 4.3.2. Focus on Radiology Entities/Institutions

Through online research, we accessed various national and international guidelines and policy directives, often aided by strategic review articles that also served a role in mediating documentation. The articles by Sarwar et al. [61] and Broder et al. [62], despite not being identified through specific keywords due to their broader focus beyond just KPIs, offer a significant overview and important insights, including references to guidelines and key documents related to KPIs in certain areas of radiology.

Sarwar et al. [61] discuss the shift in U.S. healthcare, driven by the Patient Protection and Affordable Care Act of 2010, from a volume-based to a value-based model. This transition emphasizes rewarding healthcare providers for the quality rather than the quantity of services. In radiology, current operational metrics often fail to measure value adequately. Regulatory bodies and stakeholders influence which metrics are used, with some, like the Physician Quality Reporting System, tied to financial penalties. The authors highlight a lack of metrics (including KPIs) assessing radiology’s impact on cost reduction and patient outcomes, which leads to a perception of radiology as a cost driver without recognizing its potential for cost savings and outcome improvement. They propose the development of new metrics to demonstrate radiology’s value in the evolving healthcare landscape. Broder et al. [62] emphasize the necessity for radiology departments to establish comprehensive quality and safety (QS) programs. Key principles include fostering a “just culture” and a culture of safety. QS program leaders must involve stakeholders, set clear goals, and create an effective structure. These programs should feature reliable quality assurance and patient safety systems, integrate continuous quality improvement, and prioritize patient and referring clinician experiences to enhance outcomes. Leaders must navigate common challenges in program development and management. Utilizing resources from professional societies and engaging with the radiology QS community can provide essential support for sustaining effective QS programs.

A concept paper by the European Society of Radiology [59,60] identifies five key factors that contribute to high-quality radiological practice and, indirectly, areas where KPIs are suggested to be applied

*Appropriateness of Requests:* Ensuring that imaging requests are appropriate involves compliance with guidelines, avoiding duplicate or unnecessary studies, and promoting consultations between referring physicians and radiologists. This can be facilitated through clinical decision support systems, such as ESR iGuide, to standardize and measure appropriateness.

*Radiation Protection Measures*: Adhering to the 2013/59/Euratom Directive, radiologists must prioritize non-ionizing techniques and low-dose protocols to minimize radiation exposure. Metrics for radiation protection include the presence and use of these protocols, reporting exposure levels, and ensuring education on radiation safety.

*Characteristics of the Radiology Report:* A good radiology report should be timely, correct, complete, and actionable. Structured reporting, error tracking, and regular consultations between radiologists and other specialists can improve report quality. Metrics include report completeness, accuracy, and the impact on patient management.

*Relationships between Patients and Radiology Personnel:* The quality of interactions between patients and radiology staff impacts patient satisfaction and care. Metrics include the availability of detailed preparation instructions, customer satisfaction surveys, and the existence of formal relationships with patient organizations. Policies for communicating examination results directly to patients are also considered. 

*Continuous Professional Education, Research, and Innovation:* Ongoing education and research are essential for maintaining high standards in radiology. Metrics include compliance with continuous medical education requirements, the number of subspecialty examinations, research productivity (publications, patents, and funding), and the involvement of subspecialists in consultations.

These steps emphasize patient safety and well-being, the quality of work, and the development of good relationships with patients and referring physicians. Metrics to evaluate these parameters, alongside cost assessments, could measure the overall value of radiology services. The complexity and indirect nature of some metrics pose challenges, but their development is crucial for advancing the discipline.

Interventional radiology, a subset of radiology, directly aligns with value-based healthcare as its outcomes can be more readily measured and compared to other therapeutic procedures. However, its success is interconnected with diagnostic imaging quality, necessitating the inclusion of diagnostic metrics in value assessments. 

There is also the ESR document to take into account [31], which provides an overview focusing on KPIs related to radiation protection. This document addresses various aspects within the field of radioprotection, with some implications extending to other related areas.

According to the study reported in [63], based on the ESR concept paper [64], the report of the ACR’s Economics Committee on Value-Based Payment Models [65], and the analysis conducted in [61,66,67,68], it is possible to identify the following categories with related KPIs (56 in total):

1. *Service’s Accessibility:* Measures the ease with which patients can access radiology services, including appointment availability, scheduling efficiency, and convenience for patients.
2. *Exam Prescription Adequacy:* Evaluates the appropriateness and quality of exam prescriptions based on guidelines and clinical needs.
3. *Exam Process:* Focuses on the efficiency and effectiveness of the radiology exam process from start to finish, including wait times, exam duration, and report turnaround times.
4. *Report:* Assesses the quality and completeness of radiology reports provided to referring physicians and patients.
5. *Results:* Measures the impact of radiology services on patient outcomes, treatment decisions, and satisfaction.
6. *Safety:* Focuses on patient safety measures during radiology procedures and radiation protection protocols.
7. *Contribution to the Institution:* Evaluates how the radiology department contributes to the overall institution through administrative roles, leadership positions, and certifications.

These categories cover various aspects of radiology service delivery, quality, safety, and impact on patient care and institutional effectiveness. Each category typically includes multiple specific KPIs aimed at measuring performance and driving improvements in radiology services.

The European Society of Radiology and the American College of Radiology (ACR) in the report of the 2015 global summit on radiological quality and safety specifically faced in the final recommendation the importance of the KPIs [69]. 

Key performance indicators (KPIs) are highlighted [69] as indispensable tools for documenting and measuring quality performance and safe practices in radiology. These metrics enable departments to assess their efficiency, adherence to guidelines, and overall effectiveness. 

Also, the NHS in the UK faced in detail the impact of the KPIs [70]. The section “Performance monitoring and governance processes” within document [70] delves into the operational framework governing radiology reporting within NHS trusts in England. It examines how these trusts implement and oversee key performance indicators (KPIs) designed to track the timely turnaround of radiology reports. The findings underscore a significant variation in the adoption of KPIs among trusts, revealing that some trusts lack formal indicators altogether. This disparity highlights challenges in standardizing performance metrics across the NHS.

Monitoring mechanisms, such as dashboards, are pivotal in managing these KPIs. They provide real-time insights into report turnaround times and help identify potential backlogs or delays. However, the effectiveness of these monitoring tools is sometimes hindered by technical issues related to Radiology Information Systems (RISs) and Picture Archiving and Communication Systems (PACSs). These IT challenges have occasionally compromised the reliability of data used for performance assessment.

The section emphasizes the critical importance of timely reporting in radiology services and discusses various strategies trusts employ to manage backlogs. These strategies include the re-prioritization of reports based on urgency and outsourcing of reporting tasks when internal resources are insufficient. Despite these efforts, the lack of standardized KPIs across trusts complicates national benchmarking efforts, making it challenging to compare performance and identify best practices.

Addressing these issues is crucial for enhancing operational efficiency and ultimately improving patient care within radiology services across the NHS. By promoting consistent use of KPIs, improving IT infrastructure reliability, and fostering collaboration among trusts, the document concludes that NHS can strive towards more effective and equitable radiology reporting practices nationwide.

According to the Joint Commission on the Accreditation of Healthcare Organizations (JCAHO), a key performance indicator (KPI) is defined as a critical measurement tool used to monitor and evaluate the quality of governance, management, clinical, and support functions within healthcare organizations [71]. This framework underscores the importance of integrating KPIs with strategic institutional objectives, encompassing directional change, benchmarks, targets, and specific time frames. The ability to track progress effectively is facilitated through performance dashboards or Balanced Scorecards, aligning with the guidelines emphasized in [45].

In line with these principles, [45] emphasizes the need to establish specific commissions for identifying KPIs that address strategic areas of performance within radiology departments. These strategic areas include patient safety and quality of care, where existing and potential measures directly or indirectly related to outcomes are reviewed. These measures encompass a broad spectrum:

*Patient Safety and Quality of Care:* Metrics such as the number of falls and compliance rates with hand hygiene protocols.
*Customer Service:* Assessment of patient, employee, and system-wide satisfaction with departmental services, often measured through patient satisfaction surveys.
*Operation Management and Utilization:* The evaluation of operational efficiency, including patient throughput, resource utilization, and examination durations.
*Information Technology:* Monitoring the state of information technology infrastructure, such as downtime durations for the Picture Archiving and Communication System (PACS).
*Innovation:* Development of new programs and initiatives, reflected in metrics like the number of new patent applications.
*Education:* The provision of training and credentialing for clinical and nonclinical staff, measured by the number of continuing education units awarded.
*Research:* Measurement of research productivity within the department, including the number of research papers published.
*Financial Management:* The evaluation of financial performance, such as gross revenue and technical relative value units.

These KPIs serve as essential tools for healthcare organizations to systematically evaluate and improve radiology department operations, enhancing overall efficiency, patient care quality, and stakeholder satisfaction, in accordance with the guidelines set forth in [71].

Referring to a document from the ACR [72], along with other analyses reported in [44,73], the study mentioned in [62] emphasizes the critical role of KPIs in establishing effective patient safety systems within healthcare organizations. Without specific data on quality metrics, efforts to ensure high-quality patient care are likely to falter. Therefore, a quality and safety (QS) program must carefully select KPIs that accurately monitor program progress.

This study suggests that relevant KPIs can be identified from various reputable sources, including government publications, professional societies, and other healthcare institutions. These indicators should meet criteria of relevance, achievability, and precise definition. For instance, while turnaround times (TATs) for medical examinations are readily accessible metrics, their effectiveness hinges on clear and accurate definitions. Poorly defined TATs may fail to provide actionable insights due to the complexity of the imaging process, necessitating a breakdown into more specific KPIs.

To ensure the successful tracking of performance, the chosen KPIs should align closely with both departmental and broader organizational objectives. Access to data should be convenient, and the presentation format should facilitate clear understanding. Dashboards are highlighted as an effective tool for displaying KPI data, offering a user-friendly interface that enhances data accessibility and comprehension.

An English joint document from the College of Radiographers and Royal College of Radiologists emphasizes the important role of KPIs [74] in the quality of the imaging sector and identifies various applications in the areas of Information and Support for Patients and Carers; Imaging Workforce; Scientific, Technical Support for Equipment, Facilities, and Equipment; Guidelines; Protocols; Clinical Safety; Service Organization and Liaison with Other Services; Governance; Computerized Tomography; Interventional Radiology; Magnetic Resonance Imaging; Nuclear Medicine and Molecular Imaging; and Ultrasound.

The study reported in [61], also overviewing different ACR documents and US initiatives, remarks that in radiology, traditional metrics and KPIs focus on performance management across various aspects such as report turnaround times, equipment utilization, access times, staff efficiency, and financial performance. With the emerging value paradigm, there is a growing emphasis on patient outcomes and the effectiveness of imaging services in reducing costs and improving outcomes. New metrics are geared towards measuring how imaging contributes to cost reduction and optimizing the entire care episode, including the timely diagnosis, treatment management, and immediate therapeutic responses. The study indirectly shows that ACR has evolved its stance over time to embrace these value-based metrics and outcome-driven approaches in radiology practice. Initially emphasizing technical excellence and diagnostic accuracy, the ACR now increasingly promotes the integration of imaging into broader healthcare strategies aimed at improving patient care quality and reducing healthcare costs. This evolution reflects a shift towards demonstrating the value of radiology not just through technical proficiency but also through its impact on overall patient health outcomes and healthcare system efficiency. 

Table 13 reports a sketch with the entity/source document and position on the KPIs.

#### 4.3.3. Reflection on Future Directions

This overview has underscored how emerging technologies, particularly AI in its various applications, are poised to revolutionize radiology across all its uses. AI’s potential to enhance diagnostic accuracy, streamline workflows, and support clinical decision-making marks a significant shift in the field. However, while these advancements are promising, there are evolving areas that demand the development of increasingly appropriate KPIs to ensure that these technologies are effectively integrated into clinical practice.

One critical area that requires focused attention is *the ongoing development of new diagnostic methods [75,76] and radiotherapy techniques [12,13]*. As these methods evolve, so too must the KPIs that assess their effectiveness and safety. New diagnostic technologies, which promise improved accuracy and earlier detection, will require KPIs that can evaluate their clinical utility, integration into existing workflows, and overall impact on patient outcomes.

Separately, *the integration of CAD/CAM technology* into radiotherapy [77] or in peculiar fields such as radiology in dentistry [78] presents significant challenges, particularly in the use, development, and application of materials. This integration requires KPIs that can accurately measure the quality and precision of these technologies, ensuring that they meet the high standards required in clinical environments. CAD/CAM’s role in creating customized solutions for patient care necessitates the development of specialized KPIs that can track the effectiveness of these solutions and their adherence to safety and quality standards.

*Another area of rapid evolution is tele-radiology*. As telecommunication technologies advance, tele-radiology is becoming increasingly integral to healthcare, allowing for remote diagnoses and consultation [79,80]. However, this growth brings new challenges, including the need for KPIs that can effectively measure the quality of image transmission, diagnostic accuracy, and overall service efficiency in a remote context.

Additionally, *the development of home-based radiology* represents a significant shift in how diagnostic services are delivered [81,82]. This approach brings healthcare closer to the patient but also introduces new complexities. KPIs must be developed to address the unique challenges of delivering radiology services in a home setting, including equipment portability, image quality, patient safety, and the overall effectiveness of home-based diagnostics.

All these evolving practices will have profound implications not only on the technologies themselves but also on the roles of healthcare professionals, organizational workflows, economic models, and patient experiences. As radiology embraces these new frontiers, the roles of radiologists, radiographers, and other healthcare professionals will inevitably evolve. KPIs will need to account for changes in professional responsibilities, ensuring that these roles are optimized for efficiency and patient care. Moreover, the integration of advanced technologies into daily practice will require adaptations in workflow and organizational structure, demanding KPIs that can monitor and enhance these new processes.

The *economic impact* of these advancements also cannot be overlooked [83,84]. As new technologies and practices are adopted, the cost-effectiveness of these innovations will become a critical factor. KPIs will be essential in evaluating the economic implications of new radiological methods, ensuring that they provide value while maintaining high standards of care.

*Patient-centered care* will continue to be a focal point as these technologies evolve [85]. KPIs must increasingly reflect patient outcomes, satisfaction, and overall experience, ensuring that advancements in radiology translate into tangible benefits for patients.

Overall, as radiology continues to evolve, so too must the tools we use to measure its success. The development of specialized KPIs for emerging technologies and practices is not just a necessity but a crucial step in ensuring that the field of radiology remains at the forefront of medical innovation. Through concerted efforts, these KPIs can help guide the future of radiology, ensuring that new technologies and practices are integrated effectively and contribute to the ongoing improvement in patient care. This will involve coordinated efforts across various domains—technology, professional roles, organizational dynamics, economic considerations, and patient care—to create a comprehensive and effective framework for the future of radiology.

### 4.4. Synoptic Diagram of Discussion

**Figure 3:**
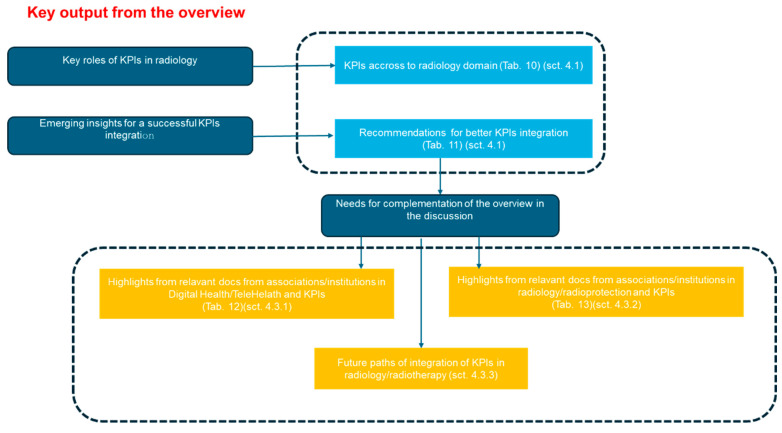
Synoptic diagram for tabular reporting of discussion.

The diagram in Figure 3 provides a highly concise sketch of the discussion, organized into tabular connections and diagrams, aligned with the overall evolution of the discourse. **Block 1** (from top to bottom) references Table 10, which highlights the role of KPIs across different radiology domains. **Block 2** references Table 11, which emphasizes the key recommendations for a better integration of KPIs in radiology within the health domain. From these recommendations in the analyzed studies, there is a unanimous call for the involvement of relevant associations and organizations in the field, summarized in **Block 4**. **Blocks 5 and 6** (moving downward from left to right) reference the highlights from relevant documents from associations/institutions in digital health/telehealth and KPIs (Table 12), and in radiology/radioprotection/radiotherapy (Table 13), respectively. The last block (**Block 7**) addresses the possible and foreseeable areas of development in this field, which were intentionally not summarized in a table or the main text. 

### 4.5. Limitations

This study was conducted by searching scientific databases for relevant research. Additionally, it was complemented by analyzing documents, reports, and guidelines from national and international scientific associations. This narrative review, aligned with the overall objectives of the general approach of this tool, highlighted emerging themes in the field. Future systematic reviews, based on advancements in scientific, medical, and technological knowledge, will be able to delve deeper into these topics. However, the research related to documents, reports, and guidelines from scientific societies and national and international associations—which are not accessible through common scientific publication databases—was limited to web searches as specified in the text. Further efforts could be directed towards establishing multi-level contacts with additional associations to obtain more comprehensive documentation.

## 5. Conclusions and Future Research Directions

### 5.1. Conclusions

This overview highlighted that KPIs are crucial for advancing the field of radiology. They encompass not only the technological evolution of imaging modalities—such as CT, MRI, and hybrid techniques—but also the integration of emerging technologies like AI and AR/VR. KPIs are essential for maintaining high standards in diagnostic accuracy, image quality, operational efficiency, and other important aspects connected to the quality in radiology in the health domain. They play a key role in ensuring that new technologies are effectively incorporated into clinical practice, thereby enhancing diagnostic capabilities and streamlining workflows.

Additionally, KPIs are also specifically vital for radiological safety, measuring adherence to protocols designed to minimize radiation exposure and protect patients. This review highlights the need for a more systematic approach to integrating KPIs into healthcare systems. Scholars have highlighted the importance of developing and validating KPIs, but practical implementation requires collaboration with healthcare providers, standardization efforts, and the development of effective implementation strategies. National and international initiatives are vital for creating uniform KPI standards, integrating advanced technologies, and fostering continuous quality improvement.

In light of evolving trends, including digital health and tele-radiology, it is imperative to consider the positions of various national and international entities. Documents from organizations like the WHO, ATA, ACP, and NHS provide valuable insights into integrating telehealth and digital health into radiology. These documents emphasize metrics for evaluating telehealth benefits, balancing clinical excellence with operational efficiency, and ensuring patient safety. Additionally, guidelines from the European Society of Radiology and the American College of Radiology outline key performance categories and KPIs crucial for assessing the quality and impact of radiological services.

Looking forward, the field of radiology must address emerging challenges such as the integration of CAD-CAM technology, home-based radiology, and advancements in diagnostic and therapeutic methods. Developing specialized KPIs to measure the effectiveness, safety, and economic impact of these innovations will be crucial. Coordinated efforts are required to create comprehensive KPI frameworks that accommodate new technologies and practices, ensuring continuous improvement in patient care.

### 5.2. Future Research Directions and Limitations 

*This review study adds significant value by offering an in-depth analysis of the integration of KPIs in radiology, focusing on key areas of interest, trends, opportunities, gaps, and areas requiring further development. It provides recommendations for improving KPI integration within the health domain.*

Ultimately, KPIs are central to the ongoing evolution of radiology, guiding the implementation of new technologies, optimizing professional roles, and enhancing patient outcomes. Addressing current limitations and leveraging these indicators effectively will help the field of radiology navigate future challenges and opportunities, advancing medical care and improving patient experiences through evidence-based practices and strategic innovations.

While this study provides a comprehensive analysis, there are some areas for future improvement:

- *Access to Documents:* The availability of certain documents was limited, particularly those not accessible through common scientific publication databases. Expanding access to diverse sources could enhance the scope and depth of future analyses.
- *Web Searches:* The use of web searches to gather documents provided a broad overview, but accessing additional detailed guidelines and practices could offer a more nuanced understanding.
- *Multi-Level Contact:* Establishing direct connections with scientific associations and organizations can enrich this review with more comprehensive documentation and insights.

Future research should focus on expanding data sources, exploring new methods of documentation retrieval, and leveraging multi-level contacts with scientific bodies to gain a fuller picture. Developing specialized KPIs tailored to new technologies and practices, and coordinating efforts across national and international bodietable ss, will further enhance the effectiveness and relevance of KPIs in radiology.

## Figures and Tables

**Table 1:** Details on the key searches.

Research Focus	Search Strategy
Basic Integration of KPIs in Radiology	*(Radiology OR Medical imaging OR Diagnostic imaging OR CT scan OR MRI OR X-ray OR Ultrasound OR Nuclear medicine OR Radiologist OR Imaging technology OR Radiographer) AND (Key performance indicators OR KPI metrics OR Performance measurement OR Healthcare metrics OR Data analytics OR Performance indicators OR Quality metrics OR Efficiency metrics OR Outcome measurement OR Performance evaluation)*
Focus on Diagnostic Imaging and KPIs	*(CT scan OR MRI OR X-ray OR Ultrasound OR Nuclear medicine) AND (Key performance indicators OR KPI metrics OR Performance measurement OR Quality metrics OR Efficiency metrics)*
Performance Measurement in Radiological Practices	*(Radiology OR Diagnostic imaging OR Imaging technology) AND (Performance measurement OR Healthcare metrics OR Data analytics OR Performance evaluation)*
Quality Metrics in Radiology	*(Radiology OR Medical imaging OR Diagnostic imaging OR Imaging technology) AND (Quality metrics OR Key performance indicators OR KPI metrics OR Outcome measurement)*
Evaluating Efficiency in Radiology	*(CT scan OR MRI OR X-ray OR Ultrasound OR Nuclear medicine OR Radiologist) AND (Efficiency metrics OR Performance indicators OR Performance evaluation OR Data analytics)*
Comprehensive Review of KPIs and Radiology	*(Radiology OR Medical imaging OR Diagnostic imaging OR CT scan OR MRI OR X-ray OR Ultrasound OR Nuclear medicine OR Radiologist OR Imaging technology OR Radiographer) AND (Key performance indicators OR KPI metrics OR Performance measurement OR Healthcare metrics OR Data analytics OR Quality metrics OR Efficiency metrics OR Outcome measurement OR Performance evaluation)*
Radiology and Outcome Measurement	*(Radiology OR Diagnostic imaging OR Imaging technology) AND (Outcome measurement OR KPI metrics OR Quality metrics OR Performance indicators)*
Impact of KPIs on Radiological Workflow	*(Radiology OR CT scan OR MRI OR X-ray OR Ultrasound) AND (Performance measurement OR KPI metrics OR Efficiency metrics OR Data analytics)*
Healthcare Metrics in Radiological Settings	*(Radiology OR Imaging technology OR Medical imaging OR Diagnostic imaging) AND (Healthcare metrics OR Key performance indicators OR Quality metrics OR Performance evaluation)*
Evaluating Diagnostic Accuracy and KPIs	*(Diagnostic imaging OR CT scan OR MRI OR X-ray OR Ultrasound) AND (Diagnostic accuracy OR Performance measurement OR KPI metrics OR Data analytics)*

**Table 2:** Key elements/points emerging in the overview of the included studies.

Study	Key Points	The Role of KPIs in the Study
*Harvey et al. (2023) [24]*	The ransomware attack on the Irish health service in May 2021 had profound consequences, particularly for CTI and its affiliated clinical trial units. KPIs, such as patient referrals and trial recruitment, plummeted by 85% and 55%, respectively, underlining the severe disruption to clinical operations. The attack compromised critical radiology and radiotherapy systems across affected hospitals, highlighting vulnerabilities in healthcare IT infrastructure. These events underscore the critical imperative for enhanced cybersecurity measures to safeguard patient care continuity and the integrity of clinical trials against future cyber threats.	A questionnaire was distributed to the units within the CTI group; this examined KPIs for a period of 4 weeks before, during, and after the attack.
*Walther et al. (2023) [25]*	In radiology, assessing the appropriateness of diagnostic imaging is crucial for quality care but lacks standardized measurement guidelines. The scoping review shows a significant variability in methodologies and criteria used across studies, highlighting the need for unified, rigorous approaches to establish reliable KPIs.	The scoping review aims to explore the definition, measures, methods, and data utilized in the analysis of appropriateness in diagnostic imaging research, a critical key performance indicator in radiology.
*Tanguay et al. (2023) [26]*	AI software is advancing quickly in radiology, with ongoing development and validation alongside new applications. However, there is a critical need for standardized protocols to assess AI software before and after it enters clinical practice. This formalization is essential to ensure patient safety, seamless integration into clinical workflows, and efficient allocation of AI development resources. Proposed frameworks aim to establish clear communication with the AI industry, equipping healthcare decision-makers and radiologists with tools to evaluate software effectively and foster a radiologist-led validation network.	The KPIs play a crucial role in this study by providing measurable metrics to assess the performance and impact of AI software in radiology.
*Wihl et al. (2021) [27]*	MDT meetings in cancer care aim to integrate comprehensive case information, but patient-related details are often under-represented, impacting decision-making. Researchers used a specific tool to assess case presentation quality and team contributions in three MDTs. Radiology information received the highest scores in MDT case discussions, emphasizing its critical role in informing clinical decisions for patients with brain tumors, soft tissue sarcomas, and hepatobiliary cancers.	The KPIs in this study serve to measure the effectiveness and quality of the decision-making processes within the MDT meetings in cancer care.
*Fayemiwo et al. (2021) [28]*	This study utilized Deep Transfer Learning models, specifically fine-tuned VGG-16 and VGG-19 Convolutional Neural Networks, to classify COVID-19 from chest X-ray images with high accuracy. This underscores their significant role in enhancing radiological diagnostics during the pandemic, surpassing existing models in accuracy and reliability.	The KPIs involve accuracy metrics such as MCC and Kappa values, assessing the effectiveness of Deep Transfer Learning models (VGG-16 and VGG-19) in classifying COVID-19 from chest X-ray images.
*Teichgräber et al. (2021) [29]*	The study developed a BSC for radiology that focuses on aligning strategic goals with the needs of referring physicians and the requirements of patients. Key components include a SWOT analysis to identify success factors, core values that emphasize high quality, and a structured value chain for radiology processes. The implementation included the creation of a strategy map to visualize cause-and-effect relationships and an automated KPI cockpit to continuously monitor and manage 18 daily and 10 annual KPIs to ensure strategic alignment and operational efficiency in radiology.	The KPIs play a central role in this study, as they serve as quantifiable metrics that measure the effectiveness and success of strategic goals in the radiology department.
*Al Shawan* *(2021) [30]*	In this study, a mixed methods approach was used to assess quality improvement and provider perceptions at King Fahd Hospital, College of the University in Khobar, Saudi Arabia. The quantitative analysis showed that there were improvements in outliers in radiology reporting after accreditation. Providers perceived accreditation positively but expressed concerns about workload and potential bias in performance metrics, highlighting the need for continuous improvement in radiology procedures during accreditation cycles.	The KPIs play a crucial role in evaluating the effectiveness of the accreditation process and its impact on quality improvement at King Fahd Hospital of Khobar University. Specifically, the KPIs were instrumental in assessing 12 quality outcomes, including those related to radiology.
*European Society of Radiology (2020) [31]*	The European Basic Safety Standards Directive 2013/59/Euratom [50] reshaped the legal framework for the use of ionizing radiation in medical imaging and radiotherapy and set strict safety and quality standards across Europe. Launched in 2014, the EuroSafe Imaging Initiative supports these objectives through comprehensive approaches such as the Guide to Clinical Audit in Radiology and the monitoring of KPIs that ensure continuous improvement and compliance with radiation protection guidelines.	The role of KPIs is to facilitate the continuous monitoring and evaluation of relevant parameters in radiology departments.
*Nason et al. (2020) [32]*	This review highlights that radiologists play a central role in the regionalization of testis cancer care by ensuring standardized imaging protocols and accurate diagnostic assessments. The introduction of KPIs in radiology is essential to monitor the quality of imaging and support multidisciplinary team discussions for optimal patient management in centralized models of care.	The KPIs play a crucial role in assessing the quality and effectiveness of regionalized testis cancer care.
*Dick et al. (2021) [33]*	In a global survey of radiology quality improvement programs conducted by the American College of Radiology’s International Economics Committee, different approaches were found in different countries. Common initiatives such as imaging adequacy and disease registries were widespread, while KPIs, peer reviews, and equipment accreditation were common, highlighting efforts to standardize and improve radiology practices worldwide. The study emphasized the need for further guidance from national and international bodies to promote consistency and optimize patient care in radiology.	In the study on quality improvement programs in radiology, KPIs play a crucial role as they were among the most frequently mentioned quality initiatives (83.3%).
*Heilbrun et al. (2020) [34]*	In this observational study, the effects of residents in diagnostic radiology on turnaround times and total costs were analyzed. It was found that turnaround times are slower, but that after-hours care by residents is faster. The presence of residents has a significant impact on radiology workflows and the efficiency of patient care, highlighting the complexity of the relationship between training costs and operational outcomes in healthcare.	In the study, KPIs were used to assess the efficiency of radiology services and compare the performance of residents and attending physicians, providing important insights into the operational dynamics of a radiology department.
*Raj et al. (2019) [35]*	Radiology played a critical role in trauma management at CWM Hospital (Fiji), with significant challenges during off-hours due to the absence of an onsite CT radiographer. The study underscores the need for improvements in trauma call processes, suggesting onsite radiographer availability and enhanced trauma team training for better patient outcomes and operational efficiency.	The KPIs such as time to the CT scan and trauma team assembly benchmarks were pivotal in assessing operational efficiency and identifying areas for improvement in trauma care at CWM Hospital.
*Pourmohammadi et al. (2018) [36]*	The study summarized the findings using the Best Fit Framework Synthesis Method, identifying efficiency/productivity, effectiveness, and finance as integral to the evaluation of hospital performance. Hospital performance management is complex and multidimensional, with each dimension having a particular importance. Selecting the most appropriate indicators is therefore the key to a comprehensive performance evaluation system.	The KPIs such as turnaround times and equipment utilization are pivotal for assessing radiology department performance within the broader institutional context.
*Obaro et al. (2018) [37]*	CTC serves as a less invasive screening method for colorectal cancer, with detection rates for advanced adenomas comparable to colonoscopy. Large-scale European trials have confirmed CTC’s efficacy in population screening. To ensure successful implementation, clinical management pathways based on initial CTC findings are crucial. Additionally, ongoing research focuses on radiologist training, quality assurance, and cost-effectiveness evaluations.	The KPIs on CTC for colorectal cancer screening help assess and optimize factors like test accuracy, uptake, quality assurance, and cost-effectiveness, crucial for evaluating its effectiveness in population screening.
*Patel et al. (2017) [38]*	In the study, the focus is on cultivating a quality culture within imaging services by integrating a quality improvement program. This program utilizes tools such as quality indicators, standard operating procedures, and Plan–Do–Study–Act cycles to identify and address process bottlenecks. The study identified seventeen KPIs spanning safety, process improvement, professional outcomes, and satisfaction. These findings underscore the significance of continuous quality improvement in diagnostic services to effectively meet the needs of both staff and end-users.	The KPIs play a critical role in assessing and monitoring the effectiveness of the quality improvement program across safety, process improvement, professional outcomes, and satisfaction domains, ensuring continuous quality enhancement in imaging services.
*Rubin et al. (2017) [39]*	RadiologyInfo.org, a comprehensive public information portal, provides over 220 multimedia resources to educate patients and raise awareness about radiology’s crucial role in healthcare. The site’s strategic planning, informed by user surveys, stakeholder interviews, and usability testing, has led to significant improvements, including website redesign, integrated video content, and the establishment of a robust affiliate network.	The KPIs serve to measure and track progress towards enhancing patient-centered content and ensuring the sustainability of RadiologyInfo.org as a vital educational resource in radiology.
*Karami and Safdari (2016) [40]*	In this study, a dashboard for medical imaging department performance indicators is developed and implemented. The process involved expert rating of indicators, determination of user interface requirements, and successful implementation of a prototype dashboard. The project identified 92 medical imaging department indicators and 53 main user interface requirements, emphasizing the significance of management information and data interoperability standards in designing effective radiology management dashboards.	The identified KPIs play a crucial role in measuring and visualizing the performance of the medical imaging department. They guide the development, implementation, and evaluation of the dashboard, ensuring effective management and operational insights.
*Schultz et al. (2016) [41]*	Beaumont’s radiation safety program integrates a diverse range of services, including diagnostic radiology, nuclear medicine, interventional radiology, various radiation therapies, and research activities. By implementing seven KPIs, Beaumont leverages objective numerical data to establish benchmarks for evaluating and improving the effectiveness and quality of its radiation safety programs over more than a decade of systematic data collection and analyses.	The KPIs outlined in this study serve as objective benchmarks for assessing and comparing the effectiveness and quality of radiation safety programs across Beaumont’s multiple-hospital system. These KPIs facilitate continuous improvement efforts and ensure compliance with regulatory standards.
*Khalifa and Zabani (2016) [42]*	King Faisal Specialist Hospital in Saudi Arabia developed 34 KPIs categorized into input, throughput, and output components to comprehensively monitor and improve ER performance. Radiology plays a crucial role in the ER performance metrics with KPIs focusing on the turnaround time for radiological services to support timely diagnoses and treatment.	The KPIs cover critical aspects such as patient acuity, wait times, staff ratios, turnaround times for essential services, and bed availability, aiming to enhance efficiency and patient outcomes in the ER setting.
*Harvey et al. (2016) [43]*	This review highlights the significance of KPIs in healthcare QA, presenting a framework for structuring KPIs, methods for their identification and customization, and strategies for analyzing and communicating KPI data to enhance process improvement. Implementing a KPI-driven QA program not only improves patient care but also enables radiology operations to showcase measurable value to healthcare stakeholders.	The role of KPIs is to enable efficient monitoring, evaluation, and improvement in radiology service quality, facilitating alignment with healthcare quality norms and enhancing overall operational effectiveness and patient care outcomes.
*Karami (2016) [44]*	This study presents a systematic approach to creating radiology dashboards, which involves identifying 92 KPIs for monitoring departmental performance and quality across services, clients, personnel, and financial aspects. The implementation of prototype dashboards showcases potential benefits in enhancing operational efficiency, productivity, and service quality within the radiology department, supporting informed decision-making and performance enhancement strategies.	The KPIs function as measurable metrics that inform the design, implementation, and evaluation of radiology dashboards, with the goal of improving operational performance, productivity, and service quality within the radiology department.
*Abujudeh et al. (2010) [45]*	The KPIs are vital metrics used to assess organizational success, tailored to reflect the unique goals and strategies of each entity. In healthcare, including radiology, these metrics are essential for enhancing patient care outcomes and guiding the implementation of best practices aimed at achieving long-term organizational goals and visions.	The KPIs play a vital role by defining, evaluating, and guiding the success and progress of healthcare organizations. Their purpose is to help achieve long-term goals and enhance patient care outcomes.
*Blakeley et al. (2008) [46]*	The study demonstrates that implementing a radiographer image reading service in a UK emergency department significantly increased image reading efficiency, reduced turnaround times, and positively impacted patient care and interdisciplinary collaboration, as evidenced by both quantitative and qualitative findings.	The KPIs were used to quantitatively assess the impact of the radiographer image reading service. These KPIs included image reading rates, turnaround times, and diagnostic accuracy.
*Koh et al. [47]*	This study emphasizes the development and monitoring of key performance indicators (KPIs) in radiography to enhance quality and safety. Starting with subjective assessments post-supervision, it evolved to include 16 measurable KPIs by 2021. Audits, with a focus on data integrity and analyses, ensure robust performance evaluation.	Evolution from subjective assessments to 16 measurable KPIs; audits ensure rigorous data collection and compliance with targets; KPIs tailored for radiography and aligned with quality and safety standards.
*Sreedharan et al. [48]*	The article discusses the implementation of KPI frameworks to evaluate institutional effectiveness in allied healthcare education. It underscores the importance of benchmarking and utilizing KPI dashboards for performance tracking across various healthcare disciplines.	Development of an institutional KPI framework; emphasis on benchmarking and utilization of KPI dashboards for tracking educational outcomes and program effectiveness.
*Lastrucci et al. [49]*	It introduces the Skills’ Retention Monitoring (SRH) tool for optimizing radiographers’ work shifts and skill management. This tool uses KPIs to enhance skill monitoring, workload management, and organizational performance, supported by continuous quality improvement measures.	Application of KPIs in optimizing work shifts and skill management; use of KPIs to track competency, workload, and organizational performance; integration with CAWI for feedback and improvement.

AI: Artificial Intelligence; BSC: Balanced Scorecard; CTC: Computed Tomographic Colonography; CTI: Cancer Trials Ireland; ER: Emergency Room; KPI: Key Performance Indicator; MCC: Matthews Correlation Coefficient; MDT: Multidisciplinary Team; QA: Quality Assurance; SWOT: Strengths/Weaknesses and Opportunities/Risks.

**Table 3:** The categorization of each one of the overviewed studies.

Study	Description	Field/Application of KPIs
*Harvey et al. (2023) [24]*	Impact of ransomware on clinical trial operations; monitoring patient referrals and trial recruitment rates using KPIs.	Cybersecurity in healthcare
*Walther et al. (2023) [25]*	Reviewing diagnostic imaging appropriateness; advocating for standardized KPIs to improve decision consistency in radiology.	Diagnostic imaging quality
*Tanguay et al. (2023) [26]*	Evaluating AI software in radiology; proposing KPI frameworks for AI performance assessment pre- and post-deployment.	AI integration in radiology workflows
*Wihl et al. (2021) [27]*	Assessing multidisciplinary team (MDT) meetings in cancer care; using KPIs to measure decision-making quality.	Cancer care quality
*Fayemiwo et al. (2021) [28]*	Using Deep Transfer Learning for COVID-19 diagnoses from chest X-rays; assessing KPIs like accuracy metrics (MCC, Kappa).	AI in pandemic diagnostics
*Teichgräber et al. (2021) [29]*	Implementing a Balanced Scorecard (BSC) for radiology; using KPIs to align strategic objectives and monitor operational efficiency.	Strategic management in radiology
*Al Shawan* *(2021) [30]*	Impact of JCI accreditation on radiology quality; using KPIs to measure radiology, reporting outliers and provider perceptions.	Quality improvement in accreditation
*European Society of Radiology (2020) [31]*	Compliance with radiation safety standards in European radiology; using KPIs to monitor safety and quality improvements.	Radiation safety and quality
*Nason et al. (2020) [32]*	Standardizing imaging protocols in centralized cancer care; using KPIs to monitor imaging quality and team discussions.	Centralized cancer care quality
*Dick et al. (2021) [33]*	Global survey of radiology quality improvement programs; highlighting KPIs like imaging adequacy and peer reviews.	Global radiology quality initiatives
*Heilbrun et al. (2020) [34]*	Impact of radiology resident training on department efficiency; using KPIs to compare resident versus attending physician performance.	Radiology training impact
*Raj et al. (2019) [35]*	Improving trauma management in Fiji; using KPIs like time to the CT scan to enhance emergency radiology services.	Emergency radiology efficiency
*Pourmohammadi et al. (2018) [36]*	Evaluating hospital performance indicators; using KPIs like turnaround times and equipment utilization in radiology departments.	Hospital performance management
*Obaro et al. (2018) [37]*	Implementing CT colonography for colorectal cancer screening; using KPIs to assess accuracy, uptake, and cost-effectiveness.	Colorectal cancer screening efficacy
*Patel et al. (2017) [38]*	Integrating a quality improvement program (QIP) in imaging services; using KPIs across safety, process improvement, and satisfaction domains.	Quality improvement in imaging services
*Rubin et al. (2017) [39]*	Impact of RadiologyInfo.org on patient education; using KPIs to measure website effectiveness and user engagement.	Patient education in radiology
*Karami and Safdari (2016) [40]*	Developing performance dashboards for medical imaging departments; using KPIs to monitor service, client, personnel, and financial aspects.	Dashboard design for radiology management
*Schultz et al. (2016) [41]*	Enhancing radiation safety programs at Beaumont Health; using KPIs to establish benchmarks and ensure compliance with safety standards.	Radiation safety program effectiveness
*Khalifa and Zabani (2016) [42]*	Improving ER performance at King Faisal Specialist Hospital; using KPIs to optimize patient flow and radiology service turnaround times.	Emergency room efficiency
*Harvey et al. (2016) [43]*	Structuring KPIs for healthcare quality assurance; using frameworks to analyze and improve radiology service quality and patient care outcomes.	Quality assurance in radiology
*Karami (2016) [44]*	Designing radiology dashboards; using KPIs to enhance departmental performance across operational efficiency and service quality.	Radiology dashboard performance enhancement
*Abujudeh et al. (2010) [45]*	Assessing KPIs in radiology quality initiatives; using metrics to guide organizational success and patient care improvements.	Quality initiatives in radiology
*Blakeley et al. (2008) [46]*	Implementing radiographer-led image reading in UK emergency departments; using KPIs to measure efficiency gains and diagnostic accuracy improvements.	Emergency radiology service improvement
*Koh et al. [47]*	Importance of using KPIs to enhance radiography performance, evolving from subjective assessments post-supervision to 16 measurable KPIs by 2021. Audits ensure rigorous data collection and analyses.	Radiography, quality improvement, safety
*Sreedharan et al. [48]*	Emphasizes KPI development for assessing institutional effectiveness in allied healthcare education. Utilizes KPI dashboards for benchmarking and tracking performance.	Allied healthcare education, performance evaluation
*Lastrucci et al. [49]*	Introduces SRH tool for radiographers to optimize work shifts, enhance skill monitoring, and improve organizational performance. Uses CAWI for feedback and emphasizes continuous quality improvement.	Healthcare workforce management, radiography

AI: Artificial Intelligence; BSC: Balanced Scorecard; ER: Emergency Room; KPI: Key Performance Indicator; MCC: Matthews Correlation Coefficient; MDT: Multidisciplinary Team.

**Table 4:** Analyzing the Impact of KPIs on Diagnostic Accuracy, Operational Efficiency, and Patient Satisfaction in Radiological Services.

Study	Contribution to Diagnostic Accuracy	Contribution to Operational Efficiency	Contribution to Patient Satisfaction
Harvey et al. [24]	N/A	KPIs assess and enhance operational resilience, being crucial post-cyberattack.	N/A
Walther et al. [25]	Calls for standardized KPIs to ensure consistency and reliability in diagnostic imaging.	N/A	Aims to improve patient care through better diagnostic imaging practices.
Tanguay et al. [26]	Establishes KPIs for evaluating AI performance, ensuring reliable diagnostic support.	Improves resource allocation and the integration of AI into workflows.	Enhances patient safety and care through reliable AI diagnostics.
Wihl et al. [27]	Measures the quality of decision-making in MDT meetings, indirectly affecting diagnostic accuracy.	Identifies gaps in information and processes, improving efficiency.	Better decision-making improves patient outcomes and satisfaction.
Fayemiwo et al. [28]	Demonstrates high diagnostic accuracy of deep learning models for COVID-19 classification.	N/A	Improved diagnostic accuracy enhances patient outcomes and confidence.
Teichgräber et al. [29]	KPIs track clinical outcomes, improving diagnostic accuracy through structured monitoring.	Enhances transparency and productivity through the Balanced Scorecard approach.	Aligns practices with stakeholder expectations, improving patient satisfaction.
Al Shawan [30]	Measures improvements in patient outcomes post-accreditation, indirectly affecting diagnostic accuracy.	Identifies operational efficiencies and challenges in quality improvement.	Enhances quality of care and patient safety through accreditation-driven improvements.
European Society of Radiology [31]	Introduces KPIs for radiation protection, affecting diagnostic accuracy and safety.	KPIs help monitor and improve radiation safety practices, enhancing operational efficiency.	Ensures that safety standards are met, improving patient trust and satisfaction.
Nason et al. [32]	Uses KPIs to track care quality and survival rates, impacting diagnostic practices indirectly.	Regionalization improves efficiency and reduces costs through centralized care.	Improves care quality and survival rates, enhancing patient satisfaction.
Dick et al. [33]	KPIs for imaging appropriateness impact diagnostic accuracy.	Highlights variability in quality programs, suggesting a need for standardized practices.	Aims to standardize practices, indirectly improving patient care and satisfaction.
Heilbrun et al. [34]	Uses turnaround time (TAT) as a KPI to measure and improve diagnostic report timing and accuracy.	Evaluates the cost and efficiency of resident training for departmental operations.	Faster and more accurate reporting improves patient care and satisfaction.
Raj et al. [35]	KPIs for trauma call times can impact diagnostic and treatment accuracy in emergencies.	Identifies inefficiencies in trauma team processes, suggesting improvements.	Enhanced trauma response times improve patient outcomes and satisfaction.
Pourmohammadi et al. [36]	KPIs assess effectiveness and safety, indirectly affecting diagnostic practices.	Evaluates efficiency, effectiveness, and financial aspects for comprehensive performance management.	Improved performance management can enhance patient care and satisfaction.
Obaro et al. [37]	KPIs like test accuracy and quality assurance improve the effectiveness of CTC in cancer screening.	N/A	Better screening effectiveness and quality assurance improve patient outcomes and satisfaction.
Patel et al. [38]	KPIs track diagnostic imaging services, indirectly impacting diagnostic accuracy.	Implements QIP to foster continuous improvement and operational efficiency.	Enhanced imaging services and quality improvement contribute to higher patient satisfaction.
Rubin et al. [39]	Uses KPIs to assess the effectiveness of a public information portal, improving radiology education.	N/A	Educating the public through improved portal content enhances patient engagement and satisfaction.
Karami and Safdari [40]	Performance dashboards with KPIs provide insights into diagnostic operations.	Enhances operational transparency and decision-making through visualized KPIs.	Improved management and transparency can lead to better patient experiences.
Shultz et al. [41]	KPIs track radiation safety, indirectly affecting diagnostic accuracy.	Monitors and improves radiation safety practices and program effectiveness.	Better safety practices improve patient trust and satisfaction.
Khalifa and Zabani [42]	KPIs for ER performance impact diagnostic and treatment accuracy in emergency care.	Improves ER operations through monitoring and managing performance indicators.	Efficient ER operations enhance patient flow and satisfaction.
Harvey et al. [43]	KPIs for QA in radiology improve diagnostic accuracy by monitoring and responding to quality issues.	Enhances operational efficiency through structured QA frameworks.	Improved QA practices lead to better patient care and satisfaction.
Karami [44]	Dashboards with KPIs provide detailed insights into diagnostic operations and performance.	Optimizes departmental performance and service quality through KPI monitoring.	Better performance and service quality improve patient satisfaction.
Abujudeh [45]	KPIs assess and improve diagnostic performance and organizational success.	Supports strategic goals and operational improvements through tailored KPIs.	Enhancing operational efficiency and care quality improves patient satisfaction.
Blakeley et al. [46]	KPIs show significant improvements in diagnostic accuracy with radiographer-led services.	Enhances ER efficiency through improved image reading services.	Improved diagnostic services and team collaboration lead to higher patient satisfaction.
Koh et al. [47]	Develops specific KPIs for radiography, improving diagnostic accuracy and system competency.	Enhances operational efficiency through rigorous KPI audits and compliance monitoring.	Better compliance and performance measures improve patient satisfaction.
Sreedharan et al. [48]	KPIs assess effectiveness in allied healthcare, indirectly affecting diagnostic accuracy.	Establishes KPI frameworks for improved institutional performance and efficiency.	Improved institutional performance can enhance patient satisfaction through better care quality.
Lastrucci et al. [49]	KPIs monitor skill retention and competencies, impacting diagnostic performance indirectly.	Optimizes work shifts and resource allocation through performance monitoring.	Better management of radiographer competencies and shifts improves patient care and satisfaction.

**Table 5:** Impact of Emerging Technologies on Development and Application of KPIs.

Study	Emerging Technologies	Key Focus	Impact on KPIs
Fayemiwo et al. [28]	Artificial Intelligence (AI)	Deep Transfer Learning frameworks for COVID-19 classification	Enhances diagnostic accuracy for COVID-19 using chest X-rays; KPIs such as Matthews Correlation Coefficient (MCC) measure AI performance.
Tanguay et al. [26]	Artificial Intelligence (AI)	Framework for evaluating AI software in radiology	Standardizes KPIs to assess AI performance, focusing on patient safety, clinical relevance, and operational efficiency.
Lastrucci et al. [49]	Artificial Intelligence (AI)	Skills’ Retention Monitoring (SRH) tool for radiographers	Enhances skill tracking and work shift optimization; AI integration can supports KPI tracking and operational performance improvements.
Nason et al. [32]	Telemedicine	Centralization of cancer care and telemedicine utilization	Improves KPIs related to patient access and timeliness by reducing geographical and administrative barriers.
Teichgräber et al. [29]	HT (Hight technology) and Standardization (Balanced Scorecard)	Development of a Balanced Scorecard (BSC) for radiology departments	Aligns strategic objectives with performance metrics; enhances transparency and accountability through standardized KPIs.
Al Shawan [30]	HT and Standardization (JCI Accreditation)	Impact of Joint Commission International (JCI) accreditation on hospital quality	Uses KPIs to track improvements in patient outcomes and operational efficiency post-accreditation.
Harvey et al. [24]	Cybersecurity	Impact of cyberattacks on cancer trial operations	Highlights the need for resilient KPIs to safeguard against disruptions and enhance operational resilience.
Walther et al. [25]	HT and Standardization	Variability in diagnostic imaging KPIs	Advocates for uniform guidelines to improve consistency and quality in radiology practices.
Shultz et al. [41]	HT in Radiation Safety	Evaluation of radiation safety programs	Utilizes KPIs to track and improve safety practices through continuous monitoring and data analyses.
Patel et al. [38]	HT and Quality Improvement Programs	Implementation of a quality improvement program (QIP) in diagnostic imaging services	Identifies measurable KPIs to foster continuous quality improvement across various domains.

**Table 6:** Emerging opportunities of the KPIs.

Study Reference	Key Findings	Emerging Opportunities
Harvey et al. [24]	Vulnerability of clinical trials to cyber threats; need for resilient KPIs.	Implementing preparedness plans and resilient KPI frameworks in clinical trial operations.
Walther et al. [25]	Opportunities for developing standardized KPIs to enhance diagnostic imaging quality and address variability in criteria.	Development of uniform guidelines and robust KPI frameworks for diagnostic imaging in radiology practices.
Tanguay et al. [26]	KPIs to evaluate AI software performance in radiology.	Defining AI software types and use cases, integrating structured KPIs for AI technologies.
Wihl et al. [27]	Multidisciplinary team (MDT) meetings in cancer care assessed KPIs. Leadership skills correlated with case presentation quality.	KPIs for MDT showed effectiveness.
Fayemiwo et al. [28]	A Deep Transfer Learning framework for COVID-19 classification using chest X-ray images has been developed.	Integrate KPIs in diagnostic protocols fostering collaborative AI research for advanced medical imaging technology.
Teichgräber et al. [29]	Introduction of Balanced Scorecard (BSC) for clinical radiology; 18 KPIs for monitoring.	Adoption of BSC frameworks for strategic alignment and continuous improvement in radiology departments.
Al Shawan [30]	Impact evaluation of JCI accreditation on quality improvement at a hospital.	Systematic use of KPIs to monitor accreditation outcomes and sustain high standards in healthcare delivery.
European Society of Radiology [31]	Development of performance indicators for radiation protection in radiology departments.	Continuous monitoring and dashboard visualization of KPIs for enhancing radiation safety practices.
Nason et al. [32]	Benefits and challenges of centralizing cancer care using KPIs.	Implementing “networks of excellence” models and leveraging telemedicine for improved cancer care outcomes.
Dick et al. [33]	Global survey on quality improvement programs in radiology; variability in KPI implementation.	Need for national and international guidance to standardize quality programs and optimize patient care in radiology.
Heilbrun et al. [34]	Cost and efficiency impacts of training radiology residents using TAT as a KPI.	Using TAT as a KPI to assess training costs and optimize departmental efficiency.
Raj et al. [35]	Evaluation of trauma call system performance using KPIs like time to the CT scan.	Continuous refinement in trauma care processes based on KPI monitoring.
Pourmohammadi et al. [36]	Synthesis of performance evaluation indicators for public hospitals.	Tailoring KPIs to specific evaluation models and organizational goals for comprehensive hospital management.
Obaro et al. [37]	Development of KPIs for ER performance; emphasis on patient flow management.	Implementing efficient ER operations through defined KPIs and patient flow metrics.
Harvey et al. [43]	Importance of KPI-driven QA programs in radiology for quality and efficiency.	Structuring QA frameworks with relevant KPIs to enhance service quality and stakeholder confidence.
Karami [44]	Design protocol for radiology dashboards using 92 identified KPIs.	Optimizing radiology performance through effective dashboard design and data-driven insights.
Abujudeh [45]	Role of KPIs in quality initiatives for radiology departments.	Implementing radiology-specific KPIs to support strategic goals and improve patient care outcomes.
Blakeley et al. [46]	Impact of radiographer-led image reading services on ER efficiency and patient care.	Enhancing ER efficiency and interdisciplinary teamwork through radiographer-led services monitored by KPIs.
Koh et al. [47]	Evolution of KPIs for radiographers; improvements in quality and safety.	Monitoring compliance and performance improvements through rigorous KPI audits and benchmarks.
Sreedharan et al. [48]	Framework for KPIs in allied healthcare education to assess institutional effectiveness.	Benchmarking and utilizing KPI dashboards for continuous improvement in allied healthcare education.
Lastrucci et al. [49]	Introduction of the Skills’ Retention Monitoring (SRH) tool for radiographers; optimizing work shifts.	Enhancing healthcare service delivery and professional development through KPI-based tool deployment.

**Table 7:** Suggestions for further research.

Study Reference	Area	Emerging Suggestions for Further Research
*Harvey et al.* [24]	Standardization of KPI Frameworks	Explore the development of universal KPI templates adaptable across diverse healthcare settings. Investigate the impact of standardized KPIs on decision-making and performance benchmarking in global healthcare contexts.
*Walther et al.* [25]	Integration of AI Evaluation	Develop standardized KPIs specifically tailored for evaluating AI algorithms in different medical specialties. Investigate the long-term efficacy and clinical outcomes of AI integration guided by robust KPI frameworks.
*Tanguay et al.* [26]	Effectiveness of Accreditation Programs	Investigate the evolving role of KPIs in JCI accreditation processes to enhance ongoing quality improvement initiatives. Assess the impact of accreditation on patient outcomes and healthcare quality using refined KPI metrics.
*Wihl et al.* [27]	Enhancement in Radiation Protection Practices	Explore innovative KPIs for real-time monitoring of radiation exposure and safety protocols. Investigate the effectiveness of new technologies in enhancing radiation safety guided by advanced KPI frameworks.
*Fayemiwo et al.* [28]	Optimization of Cancer Care Centralization	Investigate KPI-driven strategies to optimize centralized cancer care models for improved patient outcomes and healthcare efficiency. Assess the impact of standardized KPIs on equity and access to specialized cancer treatments.
*Teichgräber et al.* [29]	Global Standardization of Quality Improvement Programs	Develop a framework for globally standardized KPIs to harmonize quality improvement efforts across diverse healthcare systems. Investigate cross-country variations in KPI implementation and their impact on healthcare outcomes and patient safety.
*Al Shawan* [30]	Efficiency in Radiology Training Programs	Investigate novel KPI metrics to assess the long-term impact of radiology residency programs on healthcare quality and patient outcomes. Develop KPI-driven strategies to enhance educational efficiency and clinical preparedness in radiology.
*European Society of Radiology* [31]	Refinement in Emergency Care Protocols	Explore KPI-driven approaches to refine trauma care protocols for enhanced emergency department efficiency and patient outcomes. Investigate the role of advanced technologies in optimizing KPIs for trauma care management and response.
*Nason et al.* [32]	Enhancement in Public Hospital Management	Investigate innovative KPI metrics to enhance public hospital management strategies and improve healthcare accessibility. Assess the impact of KPI-driven interventions on healthcare equity and patient satisfaction in public hospital settings.
*Dick et al.* [33]	Advancements in Screening Technologies	Investigate the efficacy of new KPIs in evaluating emerging screening technologies for early disease detection and prevention. Develop KPI frameworks to assess the long-term impact of screening programs on population health metrics.
*Heilbrun et al.* [34]	Continuous Quality Improvement in Imaging Services	Explore novel KPI indicators to drive continuous quality improvement initiatives in imaging services. Investigate the correlation between advanced KPI metrics and enhanced patient outcomes in diagnostic imaging.
*Raj et al.* [35]	Public Engagement in Radiology Education	Investigate innovative KPIs to assess the impact of public engagement initiatives on healthcare literacy and patient outcomes. Develop KPI-driven educational strategies to enhance public awareness and involvement in radiology education.
*Pourmohammadi et al.* [36]	Development of Performance Dashboards	Investigate advanced KPI metrics for developing user-friendly dashboards in medical imaging. Assess the effectiveness of KPI-driven dashboard designs in enhancing operational efficiency and strategic planning.
*Obaro et al.* [37]	Strengthening Radiation Safety Programs	Investigate novel KPI indicators to strengthen radiation safety programs and protocols in healthcare facilities. Assess the impact of advanced KPI metrics on reducing radiation-related risks and improving patient and staff safety outcomes.
*Harvey et al.* [43]	Optimization of Emergency Room Operations	Investigate KPI-driven strategies to optimize emergency room operations and patient care pathways. Develop advanced KPI metrics to enhance emergency department efficiency and resource utilization in healthcare settings.
*Karami* [44]	Quality Assurance in Radiology Operations	Investigate innovative KPIs for quality assurance in radiology operations to improve service delivery and patient care outcomes. Develop KPI-driven strategies for addressing quality gaps and optimizing clinical workflows in radiological practices.
*Abujudeh* [45]	Dashboard Design for Operational Insights	Investigate advanced KPI metrics for designing intuitive dashboards that support data-driven decision-making in healthcare management. Assess the impact of KPI-driven dashboard designs on enhancing operational efficiency and strategic planning.
*Blakeley et al.* [46]	Promotion of Quality Initiatives in Radiology Departments	Investigate novel KPI indicators to promote quality initiatives and enhance performance metrics in radiology departments. Assess the effectiveness of KPI-driven strategies in achieving clinical excellence and patient-centered care outcomes.
*Koh et al.* [47]	Enhancement in Radiographer-Led Services	Investigate advanced KPI metrics to evaluate the impact of radiographer-led services on patient care and healthcare efficiency. Develop KPI-driven strategies to optimize interdisciplinary collaboration and enhance service delivery in radiology.
*Sreedharan et al.* [48]	Monitoring and Improving Radiography Performance	Investigate novel KPI indicators for monitoring radiography performance and optimizing clinical workflows. Develop KPI-driven interventions to enhance radiographic imaging quality and patient care outcomes in healthcare settings.
*Lastrucci et al.* [49]	Evaluation of Allied Healthcare Education	Investigate innovative KPIs for evaluating the impact of allied healthcare education on student outcomes and workforce readiness. Develop KPI-driven strategies to enhance educational effectiveness and promote continuous improvement in allied healthcare curricula.

**Table 8:** Key Recommendations for Optimizing KPIs in Radiological Services to Drive Continuous Quality Improvement.

Recommendation	Study/Studies	Key Points
*Develop and Implement Standardized KPIs*	Walther et al. [25], Tanguay et al. [26], Teichgräber et al. [29]	Standardize KPIs to ensure consistency and reliability in diagnostic imaging. Uniform guidelines help in setting clear benchmarks and goals.
*Integrate Advanced Technologies*	Fayemiwo et al. [28]	Use technologies like AI to enhance KPI precision and real-time performance monitoring. Provides more precise data for better tracking.
*Utilize Performance Dashboards*	Karami and Safdari [40], Karami [44]	Employ dashboards for real-time insights and the visualization of KPIs. Helps in identifying trends and making informed decisions.
*Adopt Quality Improvement Programs*	Patel et al. [38], Pourmohammadi et al. [36]	Regularly assess and refine KPIs through structured quality improvement initiatives. Ensures systematic addressal of performance issues.
*Focus on Patient-Centered Metrics*	Nason et al. [32], Heilbrun et al. [34]	Prioritize KPIs that impact patient outcomes and satisfaction directly. Align quality improvement efforts with patient needs and expectations.
*Establish Monitoring and Feedback Mechanisms*	Shultz et al. [41], Raj et al. [35]	Implement continuous monitoring and feedback systems to identify and address performance issues early. Crucial for ongoing improvements.
*Enhance Education and Training*	Rubin et al. [39]	Use KPIs to assess educational tools and ensure that staff are up to date with the latest practices. Effective training impacts KPI implementation and service quality.
*Emphasize Safety and Quality Assurance*	European Society of Radiology [31], Blakeley et al. [46]	Maintain high standards in safety and quality assurance. Builds patient trust and satisfaction, crucial for quality improvement.

**Table 9:** Limitations and Recommendations for Enhancing the Generalizability of KPI Studies in Radiological Services.

Study	Limitation	Potential Impact	Suggestions for Future Research
Walther et al. [25]	Focus on standardized KPIs in specific settings	Results may be context-specific	Explore applicability across diverse healthcare environments
Tanguay et al. [26]	Varied methodological approaches	Inconsistencies in KPI measurement	Develop and apply standardized KPI measurement techniques
Patel et al. [38]	Emphasis on specific KPIs and quality improvement programs	Limited view on broader KPI effectiveness	Integrate findings from a wider range of outcomes and implementations
Rubin et al. [39]	Publication bias towards positive results	May reflect an optimistic view of KPI effectiveness	Include studies with varied results for a balanced perspective
Fayemiwo et al. [28]	Focus on deep learning models for specific conditions	Results may not generalize to other diagnostic areas	Examine applicability of findings to different diagnostic technologies
Shultz et al. [41]	Specific focus on radiation safety	May not address broader KPI aspects	Broaden research to include other KPI areas beyond radiation safety
Nason et al. [32]	Conducted in high-tech, urban settings	May not be applicable to smaller or rural facilities	Assess KPI effectiveness in various healthcare settings
Harvey et al. [24]	Emphasis on KPIs post-cyberattack	Findings may be specific to cybersecurity contexts	Explore KPI effectiveness in general operational settings
Wihl et al. [27]	Measures decision-making quality in specific teams	Limited generalizability to other teams or settings	Investigate the impact of decision-making quality in diverse team settings
Karami [44]	Performance dashboards in specific contexts	May not be applicable to all radiology departments	Explore broader applications of performance dashboards across departments
Khalifa and Zabani [42]	ER performance focus	Results may be specific to emergency care	Study KPI impact in non-emergency radiological services

Notes: Focus and Scope—The limitations and suggestions for future research provided in this table aim to address the specific focus areas and methodologies of each study. This approach helps in understanding the applicability of findings across different settings and enhances the robustness of KPI-related research.

**Table 10:** KPIs Across Radiology Domains.

Aspect	Details
1. Imaging Practice Evolution	- Historical Focus: Initially centered on X-ray radiography.
	- Evolution: Expanded to include traditional radiology, CT scans, and integration with DICOM and Radiology Information Systems.
	- Current Modalities: Includes DICOM-compliant techniques such as ultrasound, MRI, gamma-ray-based methods like scintigraphy and PET scans, and hybrid methods like PET-CT and PET-MRI [51,52].
	KPIs: Track performance and quality of various imaging modalities. Key indicators include diagnostic accuracy rates, image quality, equipment uptime, and the efficiency of image acquisition and processing. KPIs ensure that advancements in imaging technologies meet high standards and that new techniques are effectively integrated into clinical practice.
2. Radiological Safety	- Radiation Exposure: Focus on minimizing exposure to patients and healthcare providers [31].
	- Quality Assurance: Includes robust protocols, equipment calibration, and regular audits.
	- Training: Continuous education on radiation protection.
	KPIs: Measure adherence to safety protocols, such as radiation dose levels, frequency of safety audits, compliance with protective measures, and staff training completion rates. KPIs help ensure that safety standards are maintained and identify areas for improvement in radiation protection practices.
3. Integration of Innovative Technologies	- Technologies: Incorporates AR, VR, and AI in radiology [52].
	- Applications: AR and VR enhance visualization for surgical planning and education. AI improves image analyses and clinical decision-making.
	KPIs: Evaluate the impact of these technologies on clinical outcomes. Indicators include diagnostic accuracy improvements, user satisfaction, integration smoothness, and the efficiency of workflows enhanced by these technologies. KPIs help assess how well innovative tools are improving diagnostic processes and patient care.
4. Role of Artificial Intelligence (AI)	- AI Integration: AI aids in image interpretation and diagnoses [53].
	- Performance Evaluation: AI systems are evaluated through KPIs.
	- Future Potential: AI could automate routine tasks and enhance overall performance.
	KPIs: Track AI performance metrics such as detection accuracy, false positive/negative rates, turnaround times, and improvements in diagnostic workflows. KPIs ensure that AI systems are effectively enhancing diagnostic capabilities and operational efficiency.
5. Digital Health and Tele-radiology	- Technologies: Adoption of secure networks for .for image sharing and tele-radiology for remote interpretation [54].
	- Benefits: Enhances access to expertise and continuity of care, especially in remote areas.
	KPIs: Measure effectiveness through indicators such as image transfer times, diagnostic report turnaround, tele-radiology consultation success rates, and patient outcomes. KPIs assess how well digital health technologies improve access to care and the efficiency of remote diagnostics.
6. Roles of Radiologists and Radiographers	- Radiologists: Interpret images, diagnose, and guide patient management [4].
	- Radiographers: Acquire images, ensure patient safety, and comfort.
	KPIs: Focus on performance metrics such as diagnostic accuracy rates, image quality, patient satisfaction, procedural efficiency, and professional development progress. KPIs help optimize the roles and effectiveness of radiologists and radiographers, ensuring high-quality patient care and professional growth.
7. Strategic Initiatives by Associations	- Role of Associations: Promote evidence-based practices, set training standards, and advocate for policy improvements [31].
	- Objectives: Support innovation, quality improvement, and collaboration.
	KPIs: Evaluate the impact of strategic initiatives through metrics such as adherence to new guidelines, research advancements, training program effectiveness, and policy changes. KPIs provide insights into the effectiveness of initiatives and their impact on the field of radiology.

**Table 11:** Summary of Strategic emerging Recommendations for Enhancing KPI Integration in Radiology.

Recommendation	Details	Action Needed	Stakeholders
*1. Standardization of KPIs*	Develop and implement uniform KPI standards to ensure consistency and reliability across institutions.	National and international radiology societies to create and enforce standardized guidelines.	RSNA, ESR, national radiological societies
*2. Integration of Advanced Technologies*	Incorporate AI to enhance KPI precision and real-time monitoring capabilities.	Support and promote initiatives for integrating these technologies through grants and research.	National health organizations, international bodies
*3. Utilization of Performance Dashboards*	Use dashboards for real-time visualization and data-driven decision-making.	Promote standardized performance dashboards and provide best practice guidelines for their implementation.	International consortia, national radiology associations
*4. Adoption of Quality Improvement Programs (QIPs)*	Implement QIPs for continuous KPI refinement and service enhancement.	Advocate for QIPs, provide training, and facilitate knowledge exchange.	National and international radiology organizations
*5. Focus on Patient-Centered Metrics*	Align KPIs with patient outcomes and satisfaction to enhance quality improvement efforts.	Develop and integrate patient-centered KPIs into clinical practice guidelines.	National health agencies, international organizations
*6. Establishment of Monitoring and Feedback Mechanisms*	Create systems for ongoing monitoring and feedback to address performance issues.	Develop systems for continuous monitoring and feedback to drive improvements.	National and international radiological societies
*7. Enhancement in Education and Training*	Provide comprehensive and up-to-date training to ensure effective KPI implementation.	Prioritize development of training programs and resources for radiologists and radiographers.	National radiology boards, international educational organizations
*8. Emphasis on Safety and Quality Assurance*	Maintain high safety and quality standards to build patient trust and improve care.	Include rigorous safety and quality assurance programs in initiatives.	National and international bodies
*9. Addressing Emerging Challenges*	Adapt KPIs to new challenges such as CAD-CAM technology for radiotherapy, home-based radiology, and tele-radiology.	Collaborate to develop and refine KPI frameworks to meet new demands and innovations.	National and international radiological communities

**Table 12:** KPIs with entities focusing on digital health.

Entity (National/International)	Position on KPIs
WHO (World Health Organization) [56]	Emphasizes demonstrating telehealth benefits during healthcare service transitions. Proposes short-term, medium-term, and long-term KPIs including increased teleconsultations, patient savings, and remote monitoring [56].
ATA (American Telemedicine Association) [57]	Focuses on balancing clinical excellence with operational efficiency in telehealth. Addresses challenges in remote care quality and suggests solutions like clinical dashboards and real-time quality reports [57].
ACP (American College of Physicians) [58]	Advocates for telehealth-specific KPIs based on in-person care quality principles. Stresses reliable performance measures tailored to telehealth environments and equity considerations [58].
NHS (National Health Service—UK) [59]	Provides KPIs for Integrated Urgent Care (IUC), integrating telehealth for optimized healthcare delivery. Metrics include call abandonment rates, response times, and remote consultations [59].
Dubai Health Authority [60]	Establishes procedures for reporting telehealth KPIs to enhance patient quality and safety. KPIs include Access and Quality metrics like patient waiting times, population coverage, and patient and staff satisfaction [60]

**Table 13:** KPIs with the entity/source document and position on the KPIs.

Entity/Brief Document Description	Position on KPIs
ACR’s Economics Committee on Value-Based Payment Models [65]	Emphasizes the integration of value-based metrics in radiology practice. This includes incentivizing quality over quantity of services, aligning with healthcare reforms such as the Affordable Care Act (ACA) to improve patient outcomes and reduce costs.
European Society of Radiology Concept Paper [64]	Identifies factors for high-quality radiological practice and suggests KPI applications. Key factors include appropriateness of imaging requests, adherence to radiation protection measures, quality of radiology reports, patient–staff interactions, and ongoing professional education and research.
ESR Document on KPIs Related to Radiation Protection [31]	Provides an overview focusing on KPIs related to radiation protection and broader implications. Discusses metrics for monitoring compliance with radiation safety guidelines, reducing radiation exposure through protocol adherence, and fostering a culture of safety within radiology departments.
NHS UK Document on Radiology Reporting [70]	Highlights variation in KPI adoption among NHS trusts and importance of timely reporting. Discusses challenges and strategies for managing radiology reporting backlogs, emphasizing the role of KPIs in improving operational efficiency and patient care.
JCAHO Guidelines on KPIs in Healthcare [71]	Defines KPIs as critical tools for monitoring healthcare quality and performance. Recommends integrating KPIs with institutional objectives to track progress effectively across clinical, managerial, and support functions within radiology departments.
College of Radiographers and Royal College of Radiologists Document (UK) [74]	Discusses applications of KPIs across various aspects of imaging services. Covers patient and caregiver support, workforce management, equipment and facility standards, clinical safety protocols, service organization, and collaboration with other healthcare services.
ACR and European Society of Radiology Global Summit on Radiological Quality and Safety [69]	Stresses the role of KPIs in enhancing quality and safety in radiology. Highlights include establishing comprehensive quality and safety programs, integrating patient feedback into service improvements, and leveraging KPIs to measure and improve radiology department performance.

## Data Availability

No new data were created or analyzed in this study. Data sharing is not applicable to this article.

## Supplementary Materials

The following supporting information can be downloaded at: https://www.mdpi.com/article/10.3390/jpm14090963/s1, Figure S1: Studies in radiology and studies in radiology with a focus on quality; Figure S2: Radiology studies published in the last five years and prior to the last five years; Figure S3: Radiology studies focused also on quality published in the last five years and prior to the last five years; Figure S4: Studies focusing on Key performance indicator in Radiology or for radiographers or for radiologists. Box S1: The proposed composite keys; Box S2: The proposed composite keys. Table S1: ANDJ checklist [86,87]. Further references on the review methodology [88,89,90].
