# Supplementary material for: Precision Metrics: A Narrative Review on Unlocking the Power of KPIs in Radiology for Enhanced Precision Medicine
Source: J Pers Med. 2024 Sep 10;14(9):963. doi: 10.3390/jpm14090963 (PMC11433247; doi:10.3390/jpm14090963)
Supplement: Supplementary file 1 [file jpm-14-00963-s001.zip › jpm-3143309-supplementary.pdf]

Journal of *Personalized Medicine*

Review

Precision Metrics: A Narrative Review on Unlocking the Power of KPIs in Radiology for Enhanced Precision Medicine

Andrea Lastrucci [1], Yannick Wandael [1], Angelo Barra [1], Vittorio Miele [2], Renzo Ricci [1], Lorenzo Livi [3], Graziano Lepri [4], Rosario Alfio Gulino [5], Giovanni Maccioni [6] and Daniele Giansanti [6,*]

[1] Department of Allied Health Professions, Azienda Ospedaliero-Universitaria Careggi, 50134 Florence, Italy; andrea.lastrucci@unifi.it (A.L.); wandaely@aou-careggi.toscana.it (Y.W.); barraa@aou-careggi.toscana.it (A.B.); riccire@aou-careggi.toscana.it (R.R.)
[2] Department of Emergency Radiology, Careggi University Hospital, Largo Brambilla 3, 50134 Florence, Italy; vmiele@sirm.org
[3] Department of Experimental and Clinical Biomedical Sciences "M. Serio", University of Florence, 50134 Florence, Italy; lorenzo.livi@unifi.it
[4] Azienda Unità Sanitaria Locale Umbria 1, Via Guerriero Guerra 21, 06127 Perugia, Italy; graziano.lepri@uslumbria1.it
[5] Facoltà di Ingegneria, Università di Tor Vergata, Via del Politecnico, 1, 00133 Rome, Italy; gulino@disp.uniroma2.it
[6] Centro Nazionale TISP, Istituto Superiore di Sanità, Viale Regina Elena 299, 00161 Rome, Italy; giovanni.maccioni@iss.it
* Correspondence: daniele.giansanti@iss.it

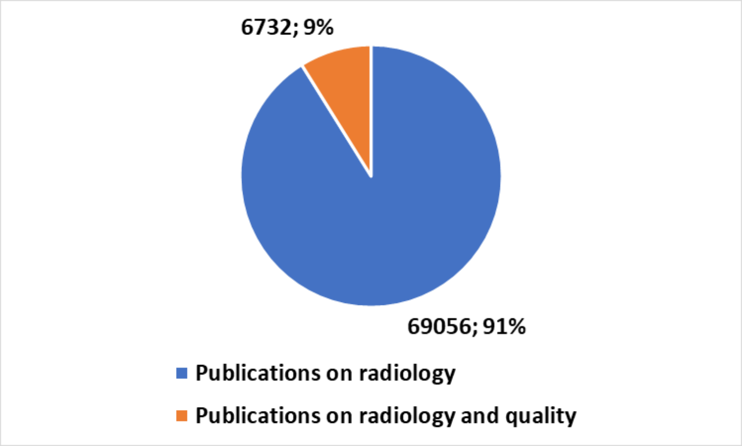

Figure S1. Studies in radiology and studies in radiology with a focus on quality.

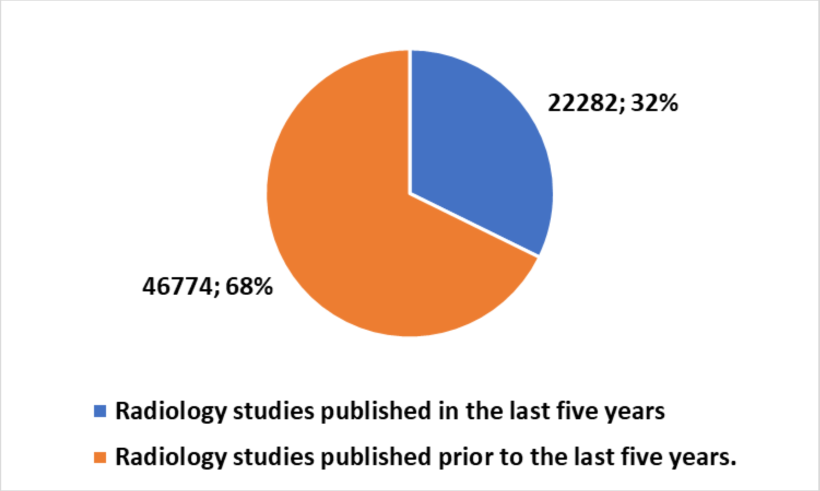

Figure S2. Radiology studies published in the last five years and prior to the last five years.

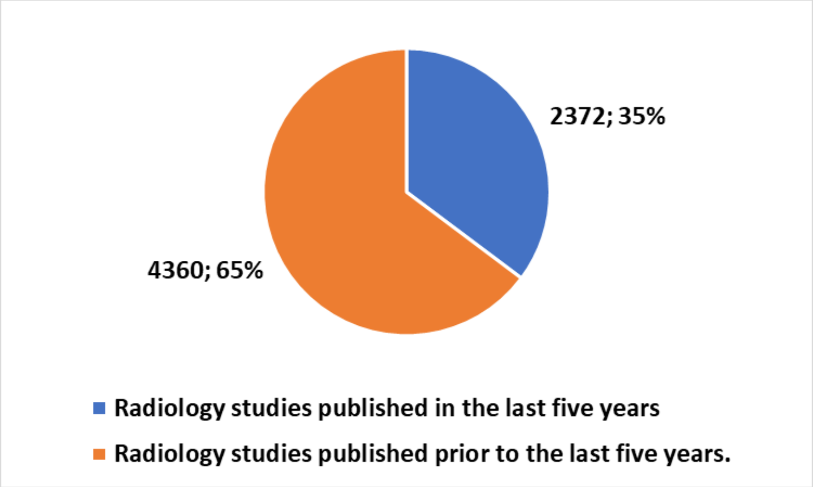

Figure S3. Radiology studies focused also on quality published in the last five years and prior to the last five years.

Box S1. The proposed composite keys.

(radiology[Title/Abstract]) .
(radiology[Title/Abstract]) AND (quality[Title/Abstract])

Box S2. The proposed composite keys.

((Key performance indicator[Title/Abstract]) OR (KPI[Title/Abstract])) AND ((radiology[Title/Abstract]) OR (radiographer*[Title/Abstract]) OR (radiologist*[Title/Abstract]))*
((Key performance indicator[Title/Abstract]) OR (KPI[Title/Abstract])) AND ((Artificial Intelligence[Title/Abstract]) OR (AI[Title/Abstract]))*

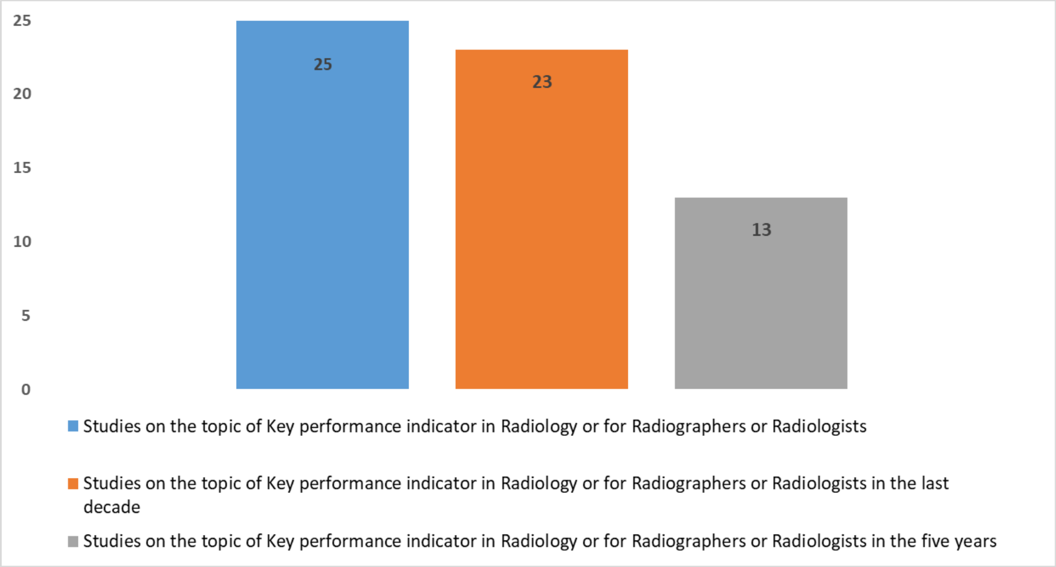

Figure S4. Studies focusing on Key performance indicator in Radiology or for radiographers or for radiologists.

Table S1. ANDJ checklist

**Section/topic**	#	**Checklist item**	**Reported on page**
**TITLE**			
title	1	Identify the report as a Narrative Review of …	**Pag. 1**
**ABSTRACT**			
	2	Provide an unstructured summary including, as applicable: background, objective, brief summary of narrative review and implications for future research, and clinical practice or policy development.	**Pag. 1-2**
**INTRODUCTION**			
Rationale/background	3	Describe the rationale for the review in the context of what is already known.	**Pag. 2-4**
Objectives	4	Specify the key question(s) identified for the review topic.	**Pag. 4-5**
**METHODS**			
Research selection	5	Specify the process for identifying the literature search (eg, years considered, language, publication status, study design, and databases of coverage).	**Pag. 5-9**
**DISCUSSION/SUMMARY**			
**Narrative**	**6**	Discuss: 1) research reviewed including fundamental or key findings, 2) limitations and/or quality of research reviewed, and 3) need for future research.	**Pag. 10-37**
Summary	**7**	Provide an overall interpretation of the narrative review in the context of clinical practice for health professionals, policy development and implementation, or future research.	**Pag. 35-36,37-50,51**

Some further useful references for the ANDJ checklist are [85–56]

S2 Integration of the methodology

The methods described in section 2.2 have been used and consolidated in different reviews. See as not exhaustive examples ref. [87–89]

S3 Analytic summaries

In the following we report the analytical summary of each one of the overviewed studies.

Harvey et al. [24] investigate the aftermath of a national cyberattack on Cancer Trials Ireland, which severely disrupted patient referrals and trial recruitment. It reports an initial 85% decrease in referrals and a 55% drop in recruitment rates. The research underscores the vulnerability of clinical trial operations to cyber threats and emphasizes the critical need for preparedness plans and resilient KPIs to safeguard against future disruptions. By examining the impact across multiple sites, the study highlights the importance of systematic data collection through KPI monitoring to assess and enhance operational resilience in healthcare settings.

Walther et al. [25] conducted as a scoping review, this study explores the methods and metrics used to assess the appropriateness of diagnostic imaging in radiology. It reveals significant variability in the criteria used across different studies and modalities. The lack of standardized KPIs emerges as a critical issue, potentially affecting the consistency and quality of clinical decision-making in radiology practices. By analyzing over 50 studies, the research calls for the development of uniform guidelines and robust KPI frameworks to enhance the reliability and appropriateness of diagnostic imaging procedures. Establishing clear KPIs could streamline assessments and improve outcomes in patient care.

Tanguay et al. [26] propose a comprehensive framework for evaluating artificial intelligence (AI) software in radiology. It addresses the growing need for standardized KPIs to assess AI performance accurately before clinical deployment. The framework outlines guidelines for defining AI software types, use cases, and their roles within clinical workflows. By emphasizing patient safety, clinical relevance, and operational efficiency, the research aims to streamline the evaluation process and optimize resource allocation. It highlights the importance of structured KPIs in ensuring the reliability, efficacy, and seamless integration of AI technologies into radiology practices, thereby supporting informed decision-making and enhancing overall healthcare delivery.

In Wihl et al.'s study [27], the focus was on assessing the effectiveness of Multidisciplinary Team (MDT) meetings in cancer care, aiming to enhance decision-making processes through the Metric for Observation of Decision-Making (MODe) tool. They used KPIs to measure decision-making quality. They examined 349 case discussions across 32 MDT meetings handling brain tumors, soft tissue sarcomas, and hepatobiliary cancers. Results indicated that while radiology information received high scores, patient-related data was often lacking. Key contributors were primarily chairs, surgeons, and oncologists, with limited input from nurses. Leadership skills positively correlated with better case presentation quality, underscoring their impact on decision outcomes.

Fayemiwo et al [28]. developed a deep transfer learning framework for COVID-19 classification using chest X-ray images. Their study showed that the fine-tuned VGG-16 and VGG-19 models achieved high accuracy rates, with VGG-16 outperforming VGG-19 in both binary (COVID-19 vs. Normal) and multiclass (COVID-19, Viral-Pneumonia, Normal) classifications. Key performance indicators such as Matthews Correlation Coefficient (MCC) demonstrated strong correlations between model predictions and true labels, highlighting the robust diagnostic capabilities of deep learning in COVID-19 diagnosis.

Teichgräber et al. [29] introduce the development of a Balanced Scorecard (BSC) specifically tailored for clinical radiology departments. The BSC framework aligns strategic objectives with internal processes and stakeholder expectations, aiming to improve service delivery and operational efficiency. By implementing 18 KPIs for daily monitoring and management, the research enhances transparency and accountability within radiology practices. The study emphasizes the role of KPIs in tracking performance metrics across key areas such as patient care, clinical outcomes, and operational productivity. By

integrating the BSC approach, radiology departments can effectively measure and optimize their performance, ensuring continuous improvement and alignment with organizational goals.

Al Shawan [30] evaluates the impact of Joint Commission International (JCI) accreditation on quality improvement initiatives at King Fahd University Hospital in Saudi Arabia. Through a mixed-methods approach, the study examines various KPIs before and after accreditation, including patient outcomes, infection rates, and operational efficiency. It identifies significant improvements in several metrics post-accreditation, reflecting enhanced quality of care and patient safety practices. Despite these gains, challenges such as observation bias and increased administrative workload are noted. The research underscores the importance of using KPIs to monitor accreditation outcomes systematically, enabling hospitals to optimize performance and maintain high standards of healthcare delivery.

European Society of Radiology [31] has developed performance indicators to enhance radiation protection practices in European radiology departments. These indicators are designed to align with the European Basic Safety Standards Directive 2013/59/Euratom (BSS Directive) and aim to improve patient and staff safety through continuous monitoring. The ESR introduced the EuroSafe Imaging Initiative in 2014, which led to the creation of the ESR Guide to Clinical Audit and its accompanying tool, known as Esperanto. KPIs are recommended for continuous monitoring and visualisation in dashboards, providing timely warnings and insights into radiation protection performance. This paper discusses various indicators and their implementation to support radiology departments in maintaining high standards of radiation safety.

Nason et al [32] focuses on the centralization of testis cancer care in Canada. It highlights the importance of key performance indicators (KPIs) and quality metrics to track and enhance the quality of care. The authors note that regionalization improves survival rates, reduces treatment morbidity, and lowers costs. However, challenges include geographical constraints, provincial healthcare administration, and patient willingness to travel. The review suggests implementing a "networks of excellence" model, similar to sarcoma care in Ontario, supported by health technology such as virtual clinics and telemedicine. Identifying and tracking KPIs is the first crucial step towards achieving these improvements.

Dick et al [33] surveyed radiology departments worldwide to determine the status of quality improvement programs, including KPIs. The survey covered 12 quality initiatives, such as imaging appropriateness, disease registries, and radiation dose monitoring. KPIs were reported by 83.3% of respondents as a common quality initiative. The study revealed significant variability in the implementation of quality programs across different countries, highlighting the need for further guidance from national and international organizations to standardize practices and optimize patient care in radiology.

Heilbrun et al [34] proposes an observational study quantifying the costs and work associated with training diagnostic radiology residents, using turnaround time (TAT) as a key performance indicator. The study found that the annual cost of a DR resident was $99,109, significantly higher than just salary and benefits. Residents participated in a substantial volume of imaging studies, and the TAT for reports was longer when residents were involved. The research underscores the financial and operational impacts of resident training on departmental efficiency and patient care outcomes, emphasizing the importance of TAT as a KPI for assessing training costs and productivity.

Raj et al [35] evaluated the implementation of a trauma call system at Colonial War Memorial Hospital in Fiji. KPIs such as time to team assembly and time to CT scan were used to assess the system's performance. The audit revealed that these benchmarks were met in only 50% of cases, highlighting areas for improvement. The study suggested that having an onsite radiographer and better trauma team training could enhance these KPIs. Continuous monitoring and refinement of the trauma call process are essential for improving patient outcomes in trauma care.

Pourmohammadi et al. [36] synthesized evidence on performance evaluation indicators for public hospitals using the Best Fit Framework Synthesis Method. The study identified three main themes: efficiency/productivity, effectiveness, and financial aspects. Efficiency/productivity indicators encompassed human resources, hospital bed usage, operating room productivity, and more. Financial indicators included profit, revenue, and cost management. Effectiveness indicators covered access, safety, quality, and responsiveness. The study emphasized the importance of selecting appropriate indicators tailored to the evaluation model and organizational goals to achieve comprehensive hospital performance management.

Obaro et al. [37] focused on CT colonography (CTC) as a screening tool for colorectal cancer (CRC). It discussed logistics, cost-effectiveness, efficiency, and advancements in CTC technology. The review highlighted key performance indicators such as test accuracy, uptake rates, quality assurance measures, and cost-effectiveness analyses. CTC offers a less invasive alternative to colonoscopy, with potential benefits in detecting advanced adenomas and extra-colonic findings. The review underscored the need for evidence-based KPIs to optimize clinical pathways and improve population-based CRC screening outcomes.

Patel et al. [38] detailed the implementation of a Quality Improvement Program (QIP) in a rural tertiary healthcare center, focusing on diagnostic imaging services. The QIP aimed to foster a culture of continuous quality improvement through a structured approach. Seventeen measurable KPIs were identified across safety, process improvement, professional outcomes, and satisfaction domains. The study utilized tools such as quality manuals, standard operating procedures, and PDSA cycles to monitor and enhance performance indicators in imaging services.

Rubin et al. [39] discussed the evolution and impact of RadiologyInfo.org, a public information portal for radiology. Key performance indicators such as website traffic, user engagement, and content optimization were used to assess the portal's effectiveness in educating the public about radiology. The study highlighted strategic planning and user-centered redesign efforts based on stakeholder feedback and usability testing. KPIs facilitated the enhancement of patient-centered content and global outreach, demonstrating effective public engagement strategies in radiology education.

Karami and Safdari research [40] focused on the development of performance dashboards for medical imaging departments (MID). Ninety-two performance indicators were identified, and user interface requirements were determined through expert consensus. The study utilized Qlikview to visualize selected KPI metrics, enhancing operational transparency and decision-making in MID management. The findings underscored the importance of data management and interoperability standards in developing effective radiology dashboards for comprehensive operational insights.

Shultz et al. [41] proposed a study from Beaumont Health System highlighting the use of KPIs to evaluate radiation safety programs across multiple hospital facilities. The program utilized objective numerical data to track and trend equipment usage, staff training, and compliance with safety protocols. KPIs provided benchmarks for assessing program effectiveness and quality over a decade, demonstrating their role in enhancing radiation safety practices in healthcare settings.

Khalifa and Zabani study [42] from King Faisal Specialist Hospital in Saudi Arabia aimed to establish a comprehensive set of KPIs for monitoring and improving ER performance. It employed a mixed-methods approach to develop 34 KPIs categorized into input, throughput, and output components of the ER patient flow model. Input indicators included patient acuity, revisit rates, and patients leaving without being seen. Throughput indicators focused on ER bed utilization, staff-to-patient ratios, and turnaround times for support services like labs and radiology. Output indicators measured boarding times and hospital bed availability, emphasizing the importance of efficient ER operations and patient flow management.

Harvey et al. [43] highlighted the role of KPIs in quality assurance (QA) within radiology operations. It emphasized that effective QA programs in radiology rely on measurable KPIs to monitor and respond to quality issues. The article proposed frameworks for structuring KPIs, methods to identify relevant indicators, and strategies for analyzing and communicating KPI data. By adopting KPI-driven QA practices, radiology departments can enhance service quality, operational efficiency, and stakeholder confidence in patient care outcomes.

Karami [44] introduced a design protocol for developing radiology dashboards, focusing on KPIs relevant to departmental performance. It identified 92 KPIs across service, client, personnel, and financial domains. The study also outlined 10 main features for dashboard design and 53 criteria for evaluating dashboard effectiveness. By implementing these dashboards, radiology managers can optimize performance, productivity, and service quality through informed decision-making and data-driven insights.

Abujudeh [45] discussed the importance of KPIs in quality initiatives for radiology departments. It emphasized the role of KPIs in evaluating organizational success and improving patient care outcomes. The study proposed radiology-specific KPIs tailored to measure performance and support strategic goals in healthcare settings. Effective use of KPIs can guide radiology practices towards enhanced operational efficiency and quality improvement initiatives.

Blakeley et al. [46] evaluated the impact of radiographer-led image reading services in an emergency department using quantitative and qualitative methods. It demonstrated significant improvements in the number of images read, turnaround times, and diagnostic accuracy following the implementation of radiographer image-reading services. The study underscored the positive effects on patient management and interdisciplinary teamwork, highlighting the valuable contribution of radiographers in enhancing ER efficiency and patient care outcomes.

Koh et al [47] discuss the importance of using KPIs to monitor and enhance performance in radiography, particularly focusing on quality and safety improvements. Initially, KPIs for radiographers were subjective post-supervised practice. The study developed specific KPIs using the Department's and Institute of Medicine's frameworks, evolving from 5 KPIs in 2013 to 16 measurable KPIs by 2021 across five broad categories with compliance targets. Audits, primarily conducted at a 3–5% sample size, ensured rigorous data collection and analysis, with full sampling for system competency and medication documentation audits. Senior radiographers conducted audits during low patient load periods, optimizing productivity. Results over nine years showed significant improvements in hand hygiene and patient ID documentation compliance, as well as in RIS PACS system competency. Overall, performance measurement fosters quality improvement, reduces waste, and provides objective benchmarks for radiographers, enhancing professional satisfaction and goal attainment.

Sreedharan et al [48] address the complexity of performance evaluation in allied healthcare education, emphasizing the importance of developing and monitoring Key Performance Indicators (KPIs) to assess institutional effectiveness. The authors conducted a literature review to establish an institutional KPI framework, highlighting the necessity for benchmarking and utilizing KPI dashboards for tracking performance. The research identifies various KPI categories essential for evaluating allied healthcare programs, stressing their role in enhancing organizational performance and ensuring quality in teaching and learning. Overall, KPIs are recognized as pivotal tools for AHIs, facilitating continuous improvement and strategic decision-making to elevate overall educational quality.

Lastrucci et al [49] highlight as optimizing work shifts in healthcare is essential for high service standards and professional growth. This study introduces the Skills Retention Monitoring (SRH) tool for radiographers, which enhances skill monitoring, workload management, and organizational performance. The SRH tool uses existing software to

track radiographers' competencies, generating weekly reports to improve resource allocation and activity management. Initial use showed significant benefits in optimizing work shifts. Feedback was collected via Computer-Assisted Web Interviewing (CAWI) during training. A continuous quality improvement approach and key performance indicators KPIs have been planned to ensure the tool's effectiveness (also opened to AI integration in perspective). The tool can be customized for various healthcare professions, supporting personalized medicine by linking competencies with patient data. Future steps include deploying the SRH tool in the Pisa hospital network and integrating AI for further enhancements. The SRH tool advances work shift optimization, enhancing healthcare service delivery and professional development for radiographers.

Supplementary references

85. https://legacyfileshare.elsevier.com/promis_misc/ANDJ%20Narrative%20Review%20Checklist.pdf
86. https://arts.units.it/retrieve/5abb6ecd-ba09-4a33-9ff6-ab8000402fc6/jcm-2839175-supplementary.pdf
87. Giansanti, D. The Regulation of Artificial Intelligence in Digital Radiology in the Scientific Literature: A Narrative Review of Reviews. Healthcare 2022, 10, 1824. https://doi.org/10.3390/healthcare10101824
88. Lastrucci A, Wandael Y, Ricci R, Maccioni G, Giansanti D. The Integration of Deep Learning in Radiotherapy: Exploring Challenges, Opportunities, and Future Directions through an Umbrella Review. Diagnostics (Basel). 2024 Apr 30;14(9):939. doi: 10.3390/diagnostics14090939. PMID: 38732351; PMCID: PMC11083654
89. Giansanti D. An Umbrella Review of the Fusion of fMRI and AI in Autism. Diagnostics (Basel). 2023 Nov 28;13(23):3552. doi: 10.3390/diagnostics13233552. PMID: 38066793; PMCID: PMC10706112

Disclaimer/Publisher's Note: The statements, opinions and data contained in all publications are solely those of the individual author(s) and contributor(s) and not of MDPI and/or the editor(s). MDPI and/or the editor(s) disclaim responsibility for any injury to people or property resulting from any ideas, methods, instructions or products referred to in the content.
